# Nanosized Porphyrinic Metal–Organic Frameworks for the Construction of Transparent Membranes as a Multiresponsive Optical Gas Sensor

**DOI:** 10.1002/smsc.202400210

**Published:** 2024-08-19

**Authors:** Francisco G. Moscoso, Juan J. Romero‐Guerrero, David Rodriguez‐Lucena, José María Pedrosa, Carolina Carrillo‐Carrión

**Affiliations:** ^1^ Center for Nanoscience and Sustainable Technologies (CNATS) Departamento de Sistemas Físicos Químicos y Naturales Universidad Pablo de Olavide Ctra. Utrera km. 1 41013 Sevilla Spain; ^2^ Institute for Chemical Research (IIQ) CSIC‐University of Seville Avda. Américo Vespucio 49 41092 Sevilla Spain

**Keywords:** absorbance, microwave, mixed‐matrix membranes, PCN‐222, porphyrin‐based metal–organic frameworks, postsynthetic metalation, sensing

## Abstract

The well‐known and excellent colorimetric sensing capacity of porphyrins, along with the exceptional structural properties of metal–organic frameworks (MOFs), make porphyrin‐based MOFs, such as PCN‐222, ideal candidates for the construction of a chemical sensor based on absorbance. However, to the best of authors’ knowledge, no high‐quality porphyrin‐based MOF gas sensors have been developed to date, most likely due to the difficulties in: 1) preparing nanosized porphyrin‐MOFs to minimize scattering in absorbance measurements; and 2) incorporating MOFs into transparent membranes for practical use. Herein, a simple and fast microwave‐assisted method for preparing high‐quality nanosized PCN‐222 crystals and their metalated derivatives PCN‐222(M) is reported to finely tune the sensing response. Next, the successful dispersion of these PCN‐222(M) nanoparticles into poly(dimethylsiloxane) to create flexible and transparent membranes is demonstrated. This integration yields a multiresponsive optical gas sensor exhibiting excellent sensitivity and the ability to discriminate between various volatile organic compounds via pattern recognition identification.

## Graphical abstract

Preparation of a multiresponsive optical gas sensor based on absorbance detection is detailed, leveraging: 1) the potential of microwave‐assisted methods for the synthesis and postmetalation of nanosized PCN‐222; 2) the exceptional sensing properties of PCN‐222 metalated with different metals for selectively modulate its response toward volatile organic compounds; and 3) the successful incorporation of PCN‐222(M) into poly(dimethylsiloxane) flexible membranes.

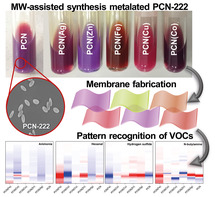

## 1 Introduction

Porphyrins are well‐known macrocyclic compounds whose extended conjugation imparts excellent electronic and optical properties.^[^ ^1^, ^2^ ^]^ These properties have been utilized, among other applications, in the design and construction of highly promising optical sensors capable of detecting and identifying a wide array of analyte types, both in solution and in the gas phase.^[^ ^3^, ^4^, ^5^, ^6^, ^7^, ^8^ ^]^ One main drawback in this development is the tendency toward molecular aggregation of these molecules,^[^ ^9^, ^10^, ^11^ ^]^ which hampers the accessibility of the analyte to the interaction site of the macrocycle. To address this problem, a number of structural components and deposition approaches have been employed, including organic molecular spacers^[^ ^12^, ^13^, ^14^, ^15^, ^16^, ^17^ ^]^ or chemical attachment to inorganic surfaces.^[^ ^18^, ^19^, ^20^, ^21^, ^22^ ^]^ However, ensuring the accessibility of the analyte to the interaction site of the porphyrin continues to be a challenging task. In this regard, integrating the porphyrin molecule as part of the structure of a metal–organic framework (MOF)^[^ ^23^ ^]^ can be an ideal solution, given the high porosity and surface area of these materials.^[^ ^24^ ^]^ Moreover, zirconium‐based porphyrinic MOFs are known to exhibit high structural, thermal, and chemical stability.^[^ ^25^, ^26^ ^]^

In general, luminescent MOFs have been successfully utilized as optical sensors^[^ ^27^, ^28^, ^29^ ^]^ over the last decade, thanks to their aforementioned properties, emerging as a robust application of these materials. In this case, the emission of the MOF, often originating from the ligand,^[^ ^30^ ^]^ from a ligand‐to‐metal charge transfer^[^ ^31^, ^32^ ^]^ or a metal‐to‐ligand charge transfer,^[^ ^33^ ^]^ is modified in the presence of the analyte, representing the typical sensing mechanism of these devices. However, porphyrin‐based MOFs exhibit a low quantum yield^[^ ^34^, ^35^ ^]^ (≈Φ≈1%),^[^ ^35^, ^36^ ^]^ leading to sensors with very low sensitivity. Another important limitation derives from the phenomenon of self‐quenching fluorescence emission, which is very common in porphyrins in high concentrations and close proximity.^[^ ^37^ ^]^ This negative effect has been reported in several porphyrinic MOFs^[^ ^38^ ^]^ due to the high concentration of porphyrins in the MOF structure, and the relatively close distance between the porphyrin units. An alternative, more suitable approach for harnessing the sensing capability of porphyrins in optical sensors involves monitoring absorbance changes in the presence of the analyte. This approach also circumvents typical drawbacks associated with fluorescence measurements, such as defects in the crystalline structure of the material,^[^ ^39^ ^]^ variations in temperature,^[^ ^40^ ^]^ or solvent effects,^[^ ^40^, ^41^ ^]^ which can cause measurements with poor repeatability, and benefits from the simplicity, cost‐effectiveness, and robustness of the absorbance measurements. Therefore, using ultrastable porphyrinic MOFs as optical sensors based on absorbance changes is an extremely practical and novel application that warrants further investigation.

In this scenario, a key requirement for obtaining high‐quality absorbance spectra of the MOF in suspension relies on having nanosized crystals with high colloidal stability to avoid light scattering. However, porphyrin‐based MOF crystals are usually produced at the micrometer scale, and reducing the crystal size to the 100–200 nm range is a challenging task. In this sense, the absorbance spectra typically shown in the literature are always affected by strong scattering,^[^ ^24^, ^42^, ^43^, ^44^ ^]^ rendering them useless for the proposed application. In addition, a pure‐phase material is highly desirable to control its structural properties in relation to the corresponding sensing capacities. In this regard, it is important to note that the synthesis of pure‐phase zirconium‐based porphyrinic MOFs is not trivial. Indeed, quite often very similar experimental conditions lead to mixed phases of different Zr‐porphyrinic MOF (i.e., PCN‐221, PCN‐222. PCN‐223, and PCN‐224),^[^ ^45^, ^46^, ^47^ ^]^ which further give rise to vast discrepancies in the material performance in diverse applications such as catalysis and sensing. Therefore, much effort should be focused on developing reproducible and robust synthetic strategies to prepare pure phase Zr‐porphyrinic MOFs to ultimately guarantee precise and reliable control of the MOF response in each specific application.

In this context, the use of microwave (MW) radiation as an energy source for the synthesis of MOFs brings together diverse beneficial aspects compared to conventional solvothermal methods. These include faster heating and a more homogeneous temperature profile, resulting in more homogeneous and smaller particles, and enabling better control over the crystalline phase‐selectivity.^[^ ^48^ ^]^ Moreover, the synthesis time can be decreased from hours or days to only a few minutes while having high yields and scaling‐up capability, which are features quite relevant from a practical point of view. Despite these advantages, which have already been demonstrated in the synthesis of various MOF types,^[^ ^49^, ^50^, ^51^, ^52^ ^]^ it is curious to realize that MW‐assisted methods are still underexploited not only in the preparation of pristine MOFs but also in their postsynthetic modification (PSM) reactions.^[^ ^50^ ^]^

In the last decade, there has been a trend in the field of chemical sensing to move from individual “key‐lock” type sensors to more versatile arrays of sensors.^[^ ^53^ ^]^ These arrays typically use nonspecific interactions to create cross‐responses that can recognize and discriminate between groups of analytes.^[^ ^3^, ^54^ ^]^ Implementing this approach with porphyrins as sensing material can be achieved by simple metalation of the porphyrin ring, which has been demonstrated as a successful method for array detection of metal‐binding species using porphyrins metalated with various metal ions.^[^ ^55^ ^]^ Specifically, this approach is particularly useful for the detection and differentiation of gaseous analytes, as many of the most toxic volatile compounds are excellent ligands for metal ions, thus producing absorbance changes upon coordination.^[^ ^56^ ^]^

The final stage in the preparation of MOF‐based gas sensors involves integrating the MOF crystals into a solid support to create a device that can be seamlessly integrated into the measurement equipment. This step poses challenges because it requires an optically inert platform capable of supporting the MOF particles while also offering accessible pores for gas diffusion and detection. Common deposition techniques like dip‐coating^[^ ^57^ ^]^ or drop‐casting do not offer precise control over film thickness and material deposition, thus hindering the effective immobilization of MOFs crystals on substrates. Other methods, such as direct crystal growth on substrates using self‐assembled monolayers, are more laborious and restrictive,^[^ ^58^, ^59^, ^60^ ^]^ requiring multiple growing cycles for the desired thickness. An interesting alternative involves incorporating MOF particles into porous polymer matrices, forming mixed‐matrix membranes (MMMs).^[^ ^61^ ^]^ MMMs offer precise control over film thickness,^[^ ^62^ ^]^ a wide range of polymer options, and functionalization possibilities, making them successful in various applications, including gas separation,^[^ ^62^ ^]^ molecular sieving,^[^ ^63^ ^]^ and gas sensing.^[^ ^32^, ^33^, ^64^ ^]^

Taking into account the aspects and challenges discussed above, we have focused our attention on this work in optimizing first a postsynthetic metalation procedure via MW irradiation to prepare a library of different metalated PCN‐222(M) (M = Ag, Fe, Zn, Cu, Co) nanoparticles capable of interacting with each analyte in a distinctive manner. Second, we have developed a simple strategy to incorporate the PCN‐222(M) particles into a polymer matrix to fabricate an array of porphyrin‐based MOFs sensors for the fast detection of a broad range of analytes with high sensitivity and exceptional selectivity. To the best of our knowledge, no high‐quality porphyrin‐based MOF gas sensors have been developed leveraging their exceptional molar absorption coefficients.

## 2 Results and Discussion

Aware that the development of optical sensing methods based on absorbance changes of MOF crystals is contingent upon the ability to prepare them at the nanometer scale, ideally with sizes equal to or smaller than 100 nm to minimize scattering issues, we faced the challenge of synthesizing PCN‐222 nanoparticles of ≈100 nm. This synthesis strategy needs to fulfill key requirements for its potential application in sensing schemes, such as 1) fast, reproducible, and scalable synthesis, crucial for potential industrial adoption, and 2) the capability to produce high‐quality PCN‐222 crystals. These crystals must be single‐pure phase, highly crystalline, and highly monodisperse to ensure a consistent sensing response. Note that the choice of the modulator greatly influences the crystallization of the MOFs; for example, PCN‐222 requires strong acidic modulators. Based on our previous experience with Zr‐porphyrinic MOFs, we selected TFA as the modulator to achieve pure phase PCN‐222. Using as a starting point a MW‐assisted method recently developed by our group for the preparation of PCN‐222 particles,^[^ ^49^ ^]^ albeit with an average size larger than optimal for absorbance measurements, we optimized the procedure with a twofold aim, to significantly decrease the particle size and increase the yield in the synthesis. After a systematic study of diverse experimental conditions, we found that smaller particles were achieved when reducing the reaction temperature, decreasing the amount of TFA used as a modulator, and increasing the initial MW power applied. Besides, decreasing the TCPP/Zr_6_ ratio but increasing the concentration of precursors led to a higher yield. Altogether, rod‐shaped PCN‐222 nanoparticles of 100 nm in length were prepared by mixing Zr_6_ clusters (76 mg, 28.4 μmol, dissolved in 4 mL DMF), TCPP (22.5 mg, 28.5 μmol, dissolved in 4 mL DMF), and TFA (100 μL), and heating this precursors mixture at 100 °C for 10 min by applying an initial MW power of 250 W. Under these conditions, the synthesis yield was 73% (based on TCPP) after purifying the PCN‐222 nanoparticles.

**Figure 1:**
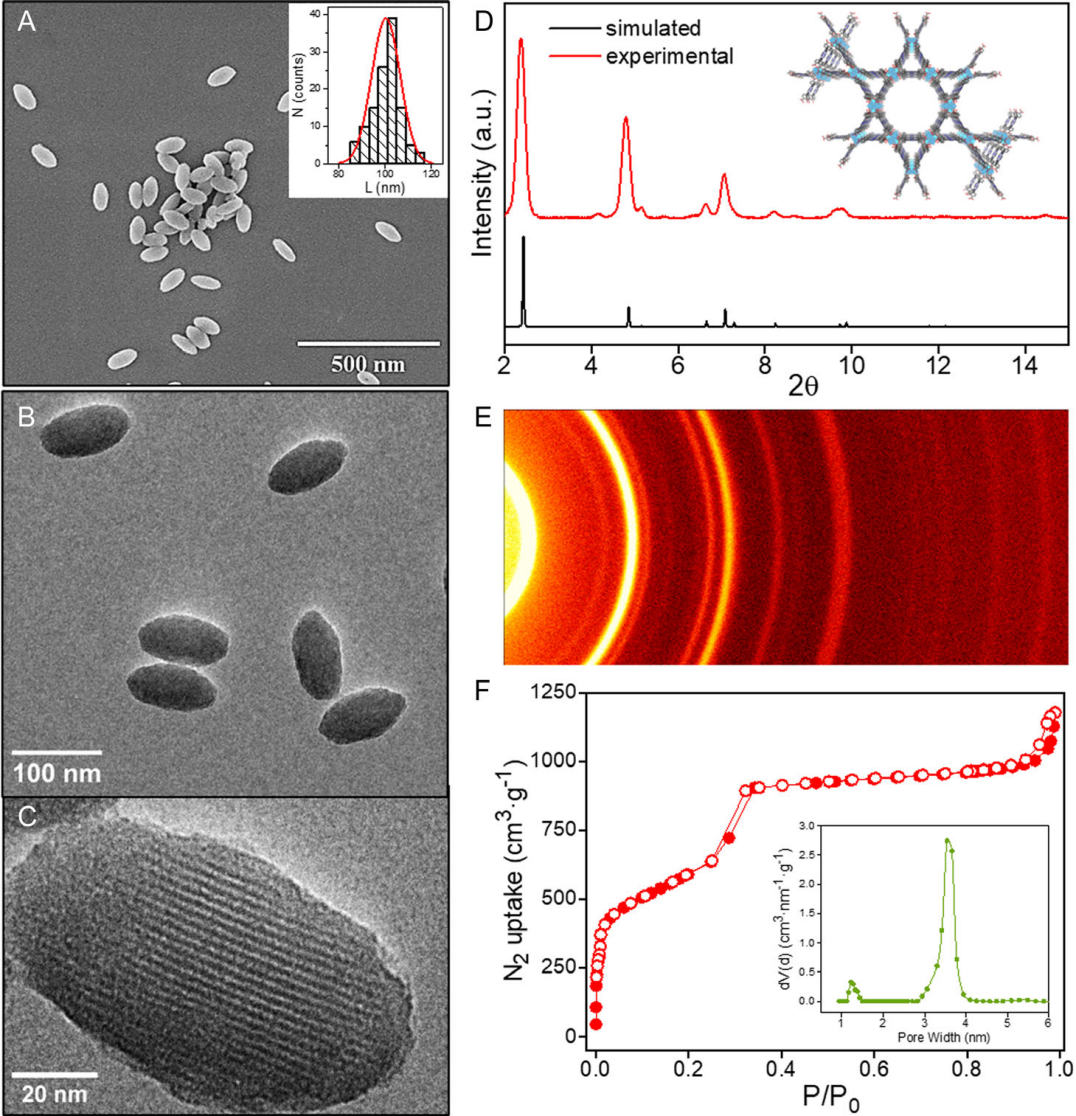
A) SEM image of PCN‐222 nanoparticles. Inset: histogram of the corresponding particle length distribution; average length *L* = 105 ± 7 nm. B) TEM image of the particles, and C) high‐resolution TEM of a PCN‐222 particle showing the oriented mesoporous 1D channels. D) PXRD spectrum of the as‐prepared PCN‐222 and the simulated pattern for comparison. Inset: 3D structure of PCN‐222 is viewed along the *c*‐axis to show the two types of pores, hexagonal mesopores and triangular micropores. E) 2525D XRD of PCN‐222. F) N_2_ isotherms (77 K) of the as‐synthesized PCN‐222 particles, and the corresponding non‐local density functional theory (NLDFT) the different PCN‐modified PDMS films were exposed to saturated vapors oftwelve analytes, i.e., 2,4‐dinitrotoluene pore size distribution analysis (inset).

Highly homogeneous rod‐shaped PCN‐222 nanoparticles with an average length of *L* = 105 ± 7 nm were obtained as revealed by scanning electron microscopy (SEM) and transmission electron microscopy (TEM) images (**Figure** **1**A–C), whereas the powder X‐ray diffraction (PXRD) associated pattern showed sharp low‐angle peaks indicative of the high crystallinity of the particles. The phase purity of the particles was confirmed by comparison of the PXRD experimental pattern with that of a predicted pattern from single crystal data (Figure 1D). The two‐dimensional X‐ray diffraction (2D‐XRD) scan along the 2*θ* axis revealed the presence of strong and well‐defined Debye rings with uniform intensity distribution, further confirming the good crystallinity and pure‐phase character of the PCN‐222 particles (Figure 1E). The Miller indices and d‐space of the reflection peaks are given in Table S3, Supporting Information. N_2_ isotherms of PCN‐222 were measured to determine the porosity of the material, which presented a type IV curve with two plateaus at relatively lower pressure and *P*/*P*_0_ = 0.3, corresponding to 1.2 nm micropores and 3.7 nm mesopores, respectively (Figure 1F). The Brunauer–Emmett–Teller (BET) area was found to be 2108 m^2^ g^−1^ and the total pore volume was 1.55 cm^3^ g^−1^ (Table S1, Supporting Information). In addition, the dynamic light scattering (DLS) measurements were carried out to evaluate the behavior of the PCN‐222 nanoparticles in solution. The hydrodynamic size of the particles dispersed in methanol was *L*_h_ = 114 nm (Figure S1 and Table S2, Supporting Information, as determined from number‐weighted size distributions) and the low polydispersity index (PDI = 0.081) confirmed the good homogeneity of the particles, in good agreement with the observations by SEM and TEM. Notably, the PCN‐222 particles presented excellent colloidal stability in methanol when stored in the fridge at 4 °C for several months, as evaluated by monitoring the changes in the hydrodynamic size over time (Figure S1, Supporting Information).

**Figure 2:**
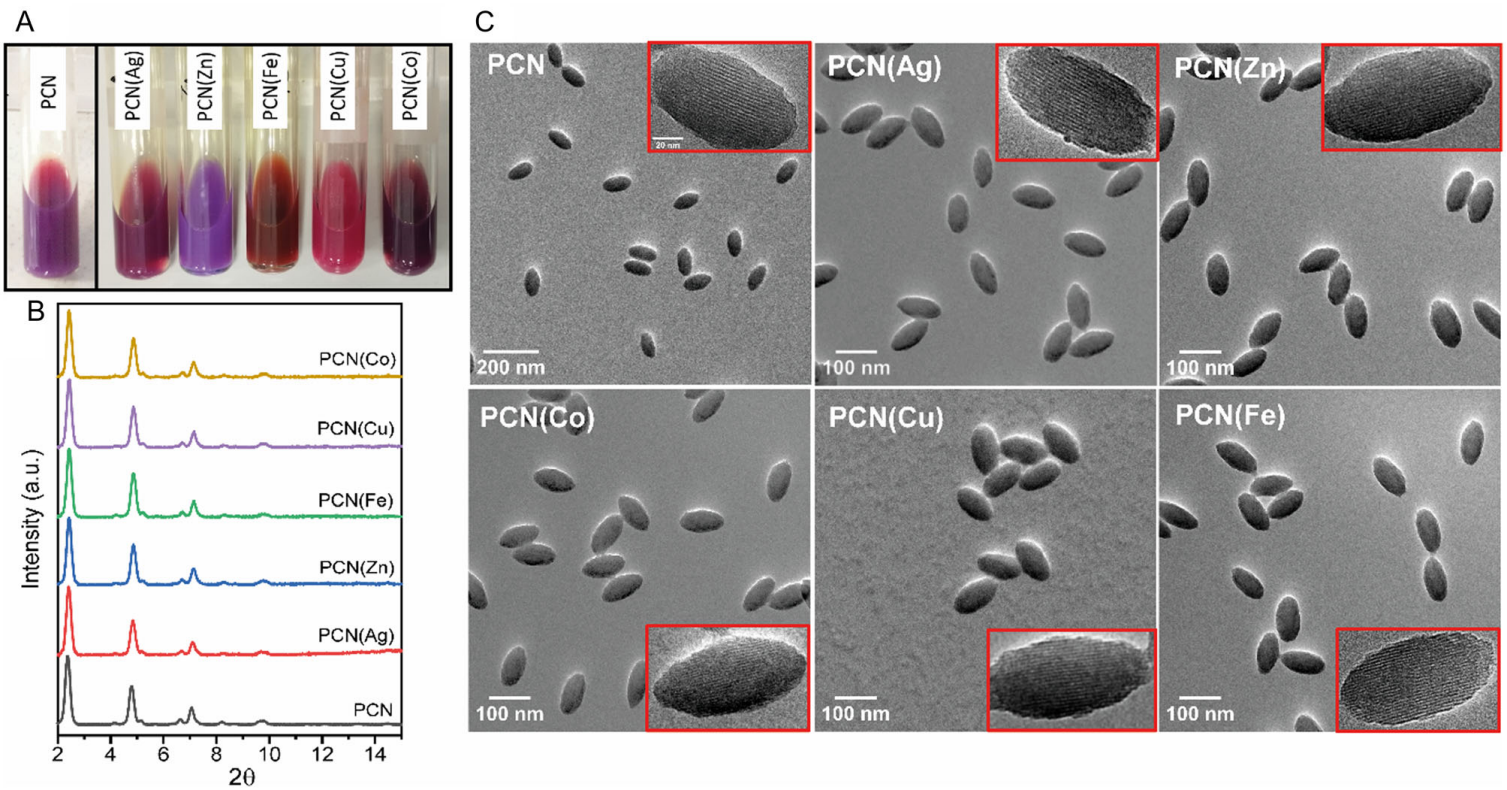
A) Photographs of dispersions of the metalated PCN(M) particles in methanol. B) PXRD patterns of the metalated particles compared to the pristine PCN‐222. C) SEM images of the metalated PCN(M) particles. Insets reveal that the ordered mesoporous channels are preserved after metalation.

With the aim of exploiting the optical properties of PCN‐222 for the construction of a sensor array with analyte recognition capacity, we proposed the metalation of the porphyrin ring with diverse metals, having access to a set of diverse metalated PCN‐222(M) nanoparticles (denoted from now on as PCN(M) for simplicity) with distinctive sensing performance. It is worth noting that the preparation of metalated PCN‐222 has been reported by using previously metalated tetrakis(4‐carboxyphenyl)porphyrin ligands, i.e., TCPP(M).^[^ ^65^, ^66^ ^]^ However, this strategy led to larger crystal sizes of the metalated MOFs in comparison with the nonmetalated counterparts. This outcome is attributed to the fact that TCPP(M) favors growth instead nucleation during the MOF polymerization process, making thus not possible to prepare nonmetalated and metalated porphyrinic MOFs with identical sizes, which is critical for the optical behavior of the MOF particles, as discussed later. To address this issue, we explored the possibility of metalation on preexisting PCN‐222 particles through a PSM approach. We exploited again the potential of the MW as an energy source to increase the kinetics and efficiency of the metalation reaction. For that, the purified PCN‐222 particles dispersed in either methanol or DMF (depending on the metal) were mixed with the metal salt solution in a MW‐vial and heated to 80 °C for 15 min by MW irradiation (see Experimental Section for details). Note that the proper choice of the solvent in this process is critical. Metalation is carried out in methanol whenever possible to prevent the presence of DMF, a coordinating solvent, within the pores, which can hinder the full access of metal ions to the porphyrin rings. Methanol is suitable for metalation with Zn(II), Cu(II), and Fe(II) because these metals are not reduced by methanol, eliminating the risk of forming metal nanoparticles. However, methanol can act as a reducing agent for Ag(I) and Co(II), leading to the formation of nanoparticles and thus preventing successful metalation of the porphyrin rings. To address this, the metalation of Ag(I) and Co(II) was carried out in DMF. After purification to remove the excess of metal precursor, the different metalated PCN‐222 particles, named as PCN(Ag), PCN(Zn), PCN(Fe), PCN(Cu), and PCN(Co) for simplicity, were redispersed in methanol (**Figure** **2**A). Note that to favor the metalation of all the porphyrins in the MOF, we used a large excess of metal ions (a molar ratio of 25:1, M:porphyrin, according to the molecular formula C_96_H_68_N_8_O_32_Zr_6_ for PCN‐222). The experimental M/Zr elemental ratio measured by TEM‐energy‐dispersive X‐ray analysis (EDX) (Figure S2, Supporting Information) was very similar in all the PCN(M) samples, of ≈0.4, which is slightly higher than the expected value (0.33; theoretical molar ratio). These results thus confirm that all the porphyrin rings were successfully metalated, and also indicate that, under the conditions used, the metalation step worked in an analogous way regardless of the metal to be incorporated into the structure. However, it seems that, in addition to those located in the porphyrin ring, there are some additional metal ions strongly adsorbed or linked to the PCN framework. One possible explanation for this observation could be that the active protons from the terminal –OH/H_2_O groups on Zr_6_ clusters may be replaced by the added metal cations forming bimetallic M/Zr clusters.^[^ ^67^ ^]^ The PCN(M) nanoparticles were also characterized by EDS elemental mapping, showing a homogeneous distribution of the metal atoms throughout the particles (Figure S3, Supporting Information).

PXRD confirmed that the metalated PCN(M) particles exhibited the same pure crystalline phase (Figure 2B and S4, Supporting Information), while TEM images indicated that the particles also retained their morphology (Figure 2C). TEM also revealed that the metalation process did not affect the ordered mesoporous channels, which were preserved in all prepared PCN(M); see insets in Figure 2C. Likewise, PCN(M) particles did not show any sign of aggregation as suggested by DLS. Comparison of intensity, volume, and number‐weighted size distributions for all the particles are presented in Figure S5, Supporting Information. As shown in Table S4, Supporting Information, the hydrodynamic sizes and PDI values were almost identical in all cases, and comparable to those of the pristine PCN‐222 particles. All these results lead us to conclude that the proposed strategy for postsynthetic metalation of PCN‐222 is both easy and versatile, being applicable to different metals. Importantly, it does not compromise the quality of the MOF particles, allowing for the tuning of their optical properties while preserving other particle features, such as size, shape, and crystallinity, unaltered. This would thus prevent misinterpretation of the results when comparing the performance of particles before and after metalation, wherein other structural aspects may unavoidably vary, as usually happens when metalated PCN‐222 particles are prepared by starting from TCPP(M) ligands. It is worth noting that the proposed postsynthetic metalation procedure is also valid for Fe(II), being not possible the preparation of PCN(Fe) from its corresponding premetalated linker TCPP(Fe).^[^ ^66^, ^68^ ^]^ This limitation is most likely due to the ability of Fe(II) cations to interact with the carboxylic groups (—COO^−^) of the TCPP linker, resulting in a cross‐linked 2D Fe‐TCPP polymer.^[^ ^69^ ^]^

**Figure 3:**
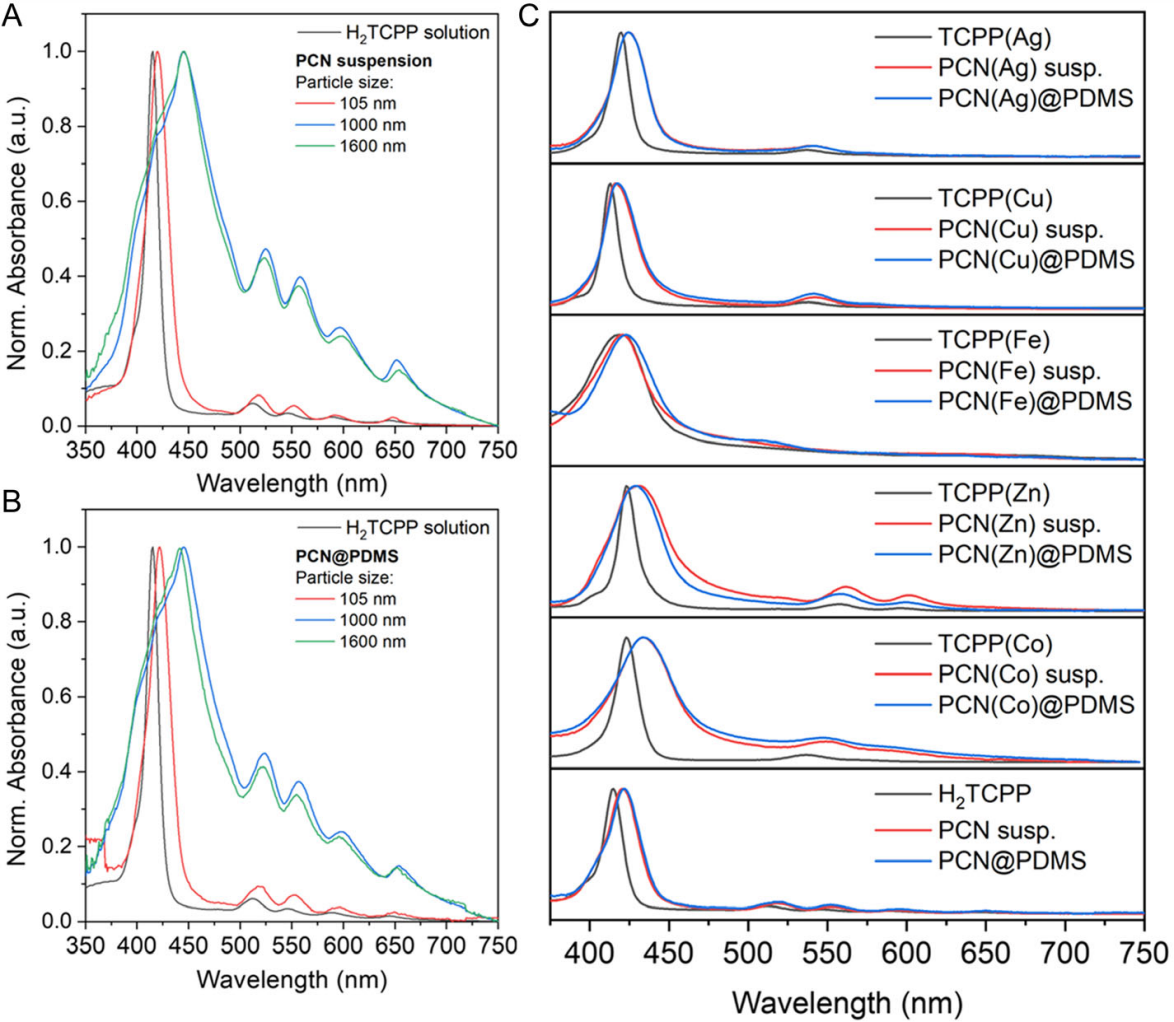
A) UV–vis spectra of H_2_TCPP (methanol solution) and PCN‐222 methanolic suspension with various particle sizes (105, 1000, and 1600 nm), B) H_2_TCPP (methanol solution) and PCN@PDMS membranes with various particle sizes (105, 1000, and 1600 nm), and C) TCPP(M) (solution), PCN(M) methanolic suspensions, and PCN(M)@PDMS films.

One of the primary objectives of this research was to mitigate light scattering in the absorbance spectra of PCN‐222 to harness the colorimetric sensing capabilities of the dye ligand in the MOF. **Figure** **3**A clearly shows that diluted methanol suspensions of PCN‐222 nanoparticles (≈100 nm in lateral size) exhibited an absorbance spectrum almost identical to that of a TCPP solution in the same solvent. This spectrum consisted of an intense Soret band at 417 nm (arising from *a*_1u_(*π*)–*e*_g_*(*π*) electronic transitions of the tetrapyrrolic core) and four weaker Q bands between 475 and 700 nm (corresponding to the *a*_2u_(*π*)–*e*_g_*(*π*) transitions).^[^ ^70^ ^]^ Specifically, there was a slight 5 nm red shift and a 10 nm broadening of the Soret band of the MOF spectrum compared to that of the free ligand. However, these changes can be deemed nearly insignificant when contrasted with the typical extensive scattering observed in the spectra of other PCNs suspensions due to the micrometric size of the obtained particles,^[^ ^24^, ^66^, ^71^ ^]^ rendering them impractical for absorbance sensing applications. Fortunately, the absence of scattering in the absorbance spectrum of our nanosized PCN‐222 suspensions as demonstrated, allowed us to exploit these particles in gas sensing utilizing this physical parameter as a response signal. Further evidence of the relevance of the particle size in the scattering observed in the absorbance spectra of PCN‐222 suspensions was obtained by preparing longer particles of ≈1000 and 1600 nm, respectively (Figure S6, Supporting Information). The corresponding absorbance spectra are included in Figure 3A. Notably, the larger particles presented Rayleigh‐type scattering, which exhibits a pronounced wavelength dependency (≈*λ* ^−4^), resulting in heightened background levels in measurements at shorter wavelengths. Consequently, they proved unsuitable for absorbance‐based optical sensing. It is noteworthy that despite their increased length, these particles remain nanometric in the other two dimensions, thereby allowing for the continued differentiation of the Soret and Q bands in their absorbance spectra. However, for selective gas sensing based on absorbance alterations, well‐resolved spectra are essential to clearly attribute these changes to specific analytes. In this regard, solid‐state effects within the crystal also vary with particle size and are known to impact spectral properties.^[^ ^72^ ^]^ Specifically, alterations in band maximum and width were observed with increasing particle size. While nanosized PCN‐222 suspensions exhibited only minimal broadening (FWHM_TCPP_ = 14.7 nm, FWHM_PCN‐222_ = 25.6 nm) and a slight red‐shift (Δ*λ* = 5 nm) of their Soret band compared to H_2_TCPP,^[^ ^50^, ^51^, ^52^ ^]^ larger particles experienced more significant band broadening and shift due to more pronounced solid‐state effects (FWHM_1000_ = 90.2 nm, FWHM_1600_ = 90.3 nm, Δ*λ*_1000_ = 30 nm, Δ*λ*_1600_ = 31 nm).

The next step in constructing a PCN‐based sensing device involved integrating the MOF nanocrystals into a transparent and porous poly(dimethylsiloxane) (PDMS) membrane. The resulting MMMs appeared highly homogeneous and free from particle aggregation, thanks not only to the small size of the PCN‐222 crystals but also to their exceptional colloidal stability in suspension that allowed their incorporation as individual particles and not as aggregates. As depicted in Figure 3B, there were no discernible differences in the absorbance spectra between the PCN‐222 particles in suspension (Figure 3A) and those incorporated into the polymer matrix. Furthermore, the PDMS matrix itself exhibited negligible absorbance across the measured wavelength range, as indicated by its absorbance spectrum in Figure S7A, Supporting Information. This confirms that the PDMS does not interfere with the optical properties of the PCN‐222, ensuring that the observed absorbance is solely attributable to the MOF nanocrystals. Visually, as shown in Figure S7B and S7C, Supporting Information, the PDMS membrane is colorless and transparent, while the PCN@PDMS membrane displays a slight coloration due to the presence of the PCN‐222. Metalated PCN‐222 were also interrogated by absorption UV–vis spectroscopy, yielding similar results to those of free PCN‐222, as depicted in Figure 3C. Specifically, PCN(M) suspensions also exhibited a slight broadening and red shift of the Soret band compared to the ligand solution. However, the corresponding absorbance spectra did not show any appreciable evidence of light scattering. Additionally, the spectra of PCN(M)@PDMS membranes were almost identical to those of the MOF in methanol suspension in all cases. Closer inspection of the spectra in Figure 3C revealed further evidence of the successful metalation of the porphyrins in the PCN‐222 through the proposed postsynthetic method. First, the four Q‐bands in the PCN‐222 spectrum coalesced into two Q‐bands in all the PCN(M) spectra due to the increase in symmetry from *D*_2h_ to *D*_4h_ upon metalation of the porphyrin in the MOFs. Additionally, this spectral profile of the PCN(M) suspensions remained unaltered after mild acid treatment, specifically the addition of trifluoroacetic acid (TFA:porphyrin molar ratio 10:1), as depicted in Figure S8, Supporting Information. In contrast, the PCN‐222 spectrum partially experienced the typical changes associated with the protonation of non‐metalated porphyrins. Specifically, a new band (Soret) appeared at 445 nm, and there was an increase in the Q band at ≈650 nm.

**Figure 4:**
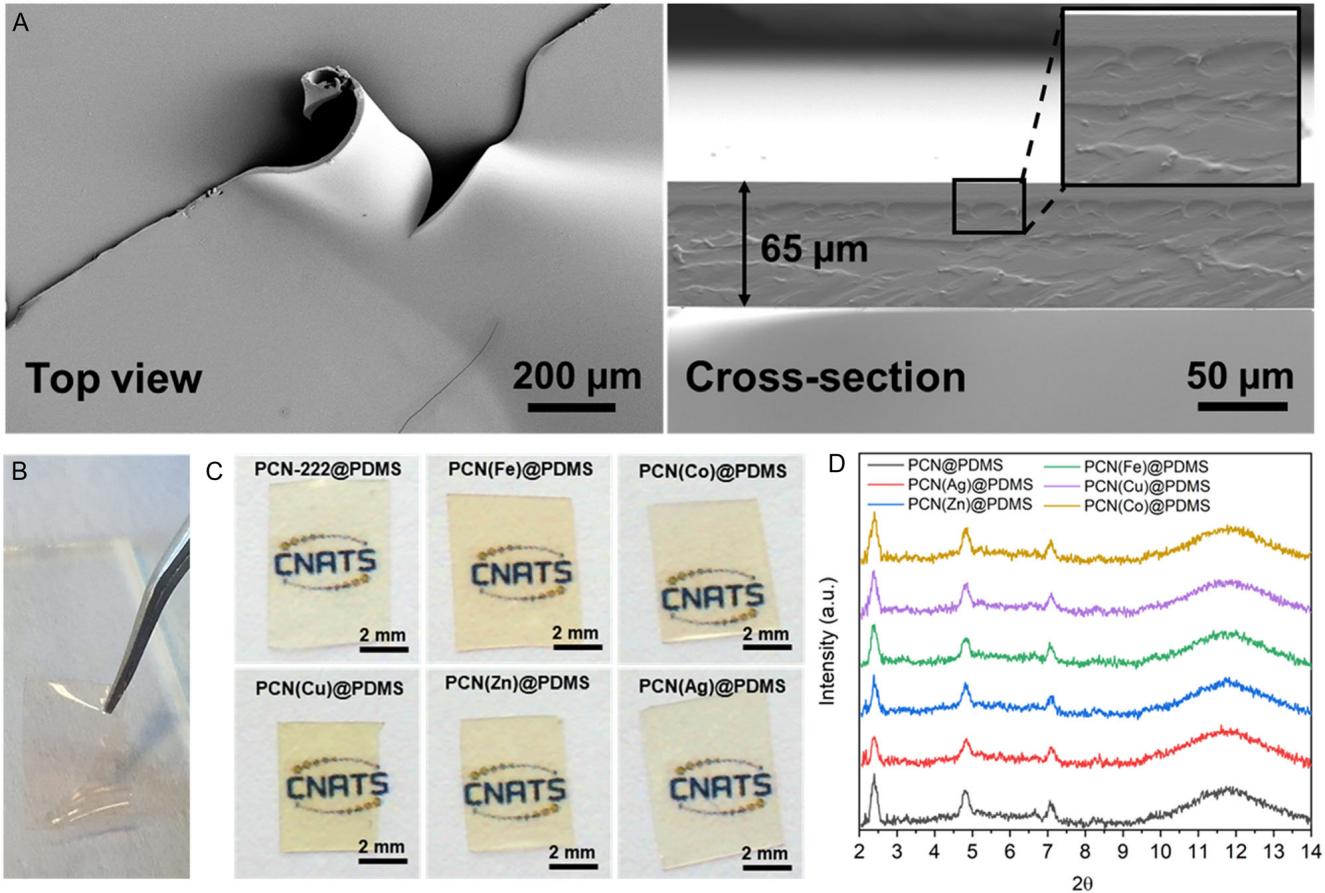
A) Top view and cross‐section SEM images of a PCN@PDMS film. B) Photograph showing the flexibility of the PCN@PDMS film. C) Photographs showing the transparency and color of different PCN(M)@PDMS films. D) GAXRD of PCN@PDMS and PCN(M)@PDMS films.

The high quality of the absorbance spectra of the PCN@PDMS films, as depicted in Figure 3, was also facilitated by the optimization of our deposition method in PDMS.^[^ ^24^, ^31^, ^33^, ^44^, ^54^, ^73^ ^]^ Specifically, we initially dispersed the MOF nanocrystals in methanol and subsequently added the PDMS precursors. This approach enabled us to achieve a significant improvement in uniformity and casting abilities while also reducing particle aggregation and obtaining membranes free of defects. Top‐view SEM images of PCN@PDMS films demonstrate that the MOF particles were fully embedded into the PDMS polymer matrix (**Figure** **4**A, left), while cross‐section images (Figure 4A, right) reveal a consistent thickness of 65 μm. Similar results were obtained for the PCN(M)@PDMS membranes. The high transparency of the flexible and slightly colored PCN(M)@PDMS membranes could be appreciated by the naked eye, as revealed by the photographs in Figure 4B,C. Grazing‐angle X‐ray diffraction (GAXRD) patterns of the corresponding membranes also demonstrate that the crystallinity of the PCN particles was preserved once embedded in the polymeric film, since some of their characteristic diffraction peaks could be observed on the noisy signal caused by the amorphous PDMS (Figure 4D) characterized by an amorphous phase around 12° (2*θ*).

The gas‐sensing properties of PCN(M)@PDMS films were studied by analyzing the spectral changes undergone by the porphyrin‐based MOFs in the UV–visible region upon their exposure to vapors of twelve different analytes that belong to different target groups, including explosives such as nitroaromatic compounds (2,4‐dinitrotoluene); inorganic acids (hydrogen chloride, hydrogen sulfide), and bases (ammonia); and volatile organic compounds such as alcohols (ethanol), aldehydes (hexanal), amines (N‐butylamine), nonpolar solvents (toluene), and polar solvents (acetone, tetrahydrofuran, chloroform, dichloromethane). The corresponding absorption spectra before and after exposure to the different analytes were analyzed, showing noticeable changes as depicted in Figure S9–S20, Supporting Information.

**Figure 5:**
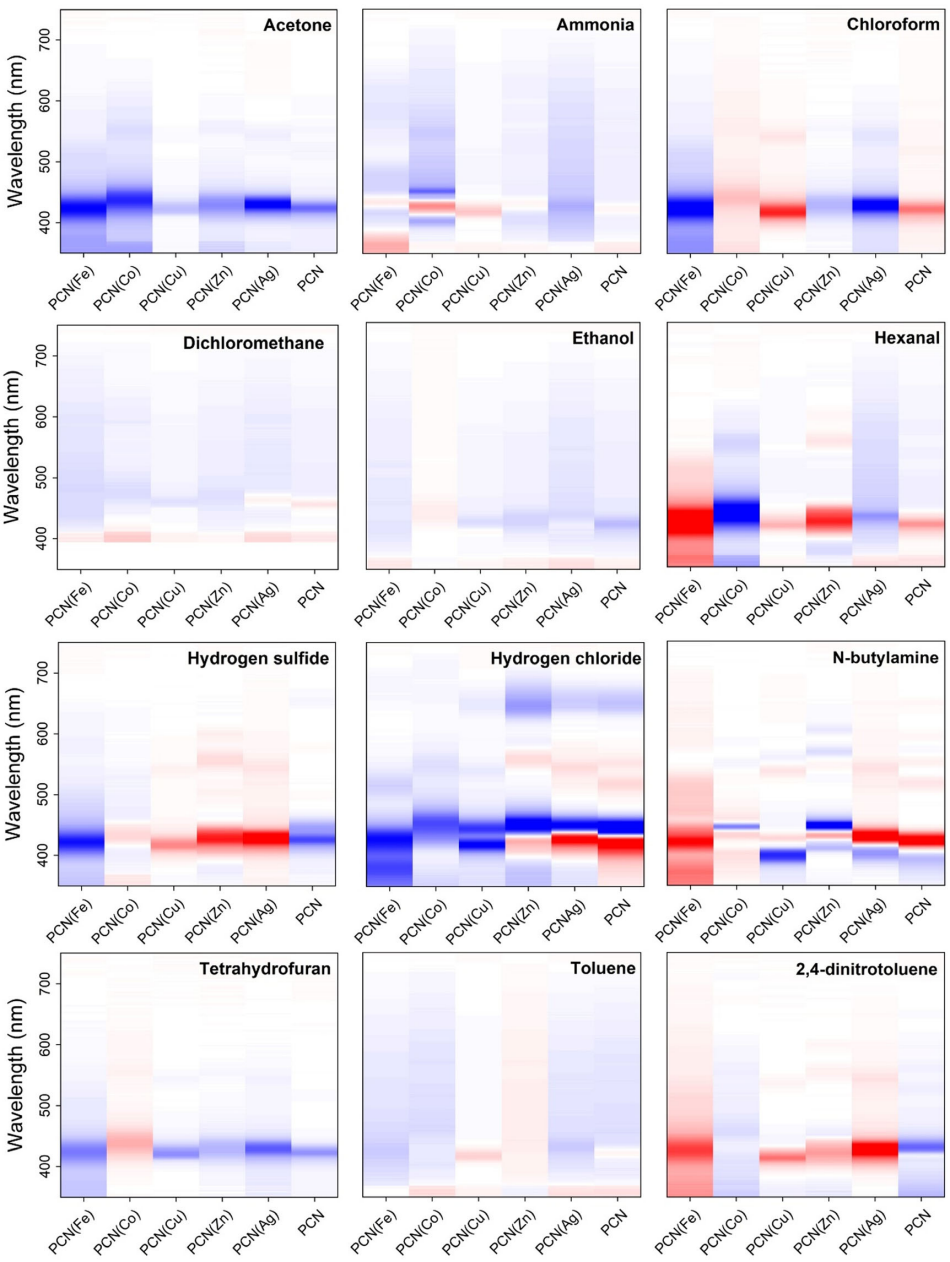
Identification patterns obtained for acetone, ammonia, chloroform, dichloromethane, ethanol, hexanal, hydrogen chloride, hydrogen sulfide, N‐butylamine, tetrahydrofuran, toluene, and 2,4‐dinitrotoluene. The negative difference between exposure and nonexposure absorbance spectra trends toward red and the positive difference trends toward blue (see further details in the text).

To facilitate the visualization of the spectral changes, the exposure spectrum was subtracted from the preexposure spectrum and normalized to the maximum absorbance of the preexposure spectrum. The difference spectra of the 6 PCN(M)@PDMS films were grouped for each analyte and converted into an *m* × *n* matrix, where *m* is the wavelength and *n* is the number of MOFs included in this study (*n* = 6). Finally, the matrix was represented in a heat map, creating recognition patterns for each analyte that are depicted in **Figure** **5**. Each pattern consists of 6 columns corresponding to the six MOFs (PCN‐222 and five PCN(M)), and each column shows the difference between the corresponding absorption spectra before and after exposure. This difference is represented on a color scale from red to blue, where a negative difference trends toward red and a positive difference trends toward blue. If no change is observed, the color remains white. The wavelength (*Y*‐axis in the patterns) grows from down to top in each column. Through this representation, the band shifts and absorbance changes produced by the different analytes (higher values in the difference spectra) can be easily distinguished, giving specific information about the compound to be identified. As depicted in Figure 5, each analyte exhibited a distinct pattern that distinguishes it from the others, facilitating the visual identification of all analytes tested. Although certain patterns may share similarities, such as ethanol and dichloromethane, they were not identical. Upon closer examination, N‐butylamine, hydrogen chloride, hexanal, and acetone were identified as the analytes that elicit the strongest response, particularly when exposed to PCN(Ag)@PDMS, which appears to be the most sensitive material. Overall, the sensor array comprised of PCN@PDMS and PCN(M)@PDMS has demonstrated promising capabilities for gas sensing screening. The response is highly selective, enabling the identification of various types of analytes.

The stability of PCN@PDMS and PCN(M)@PDMS films was confirmed by comparing their absorbance spectra and X‐ray diffractograms at preparation and after 3 months. Figure S21, Supporting Information, demonstrates that the MOFs remained crystalline even after 3 months, with no changes observed in the absorption spectra. This indicates that the structure remained unaltered and free of degradation, which is a key for the long‐term storage of PCN(M)@PDMS films and consequently for actual application potential.

Further characterization of the sensing performance of the PCN@PDMS film was obtained by studying the kinetics of their spectral response when exposed to HCl vapors. This analyte is detected through a well‐known mechanism involving the protonation of the porphyrin ring. This protonation results in a brown‐to‐green color change that is clearly visible to the naked eye, along with well‐defined spectral changes consisting of a strong red‐shift of the Soret band and the reduction of the number of Q bands from 4 to 2 (for symmetry reasons), with a specific enhancement of the band at 650 nm. As shown in Figure S22, Supporting Information, a rapid response to the injection of HCl gas was observed by the decrease in absorption at 417 nm, caused by the shift of the Sored band, and the increase of the absorbance intensity at 650 nm. The response rate was calculated from the kinetic curve using the parameters *t*_50_ and *t*_90_, defined as the time required to reach 50% and 90% of the total absorbance change, respectively. The *t*_50_ and *t*_90_ were 3.8 and 8.45 s, respectively, highlighting the high speed of the response. Furthermore, this rapid change indicates that the MOF, even when embedded in the PDMS film, remains readily accessible to the incoming gas, with no pore blockage limiting the diffusion of the gas to the active sites of the sensor. Despite the fast and intense sensing response, complete desorption of the analytes from the membrane within a reasonable timeframe was not possible, making the membranes disposable rather than reusable. On a positive note, a functional membrane in an optical device would contain only a few micrograms of MOF. Moreover, considering that any variation in the preexposure spectra from reused membranes could lead to incorrect recognition patterns, single‐use MOF membranes are preferable. They ensure the reliability and reproducibility of the sensing response, maintaining high selectivity by using fresh membranes for each sensing event.

## 3 Conclusion

Rod‐shaped PCN‐222 nanoparticles with high crystallinity and homogeneity have been successfully synthesized and further metalated with different metals in a simple and efficient manner through a MW‐assisted method, which allowed tuning the optical properties and sensing capabilities of the PCN‐222 as a function of the metal incorporated into the porphyrin.

Additionally, we demonstrated that these nanoparticles, once embedded into PDMS films, presented an excellent optical quality, with almost no light scattering and high transparency, enabling the detection of gaseous analytes in a selective way by combining the information obtained from the absorption spectra before and after the exposure of different analytes. The sensor array formed by PCN@PDMS and PCN(M)@PDMS (M = Fe, Co, Cu, Zn, Ag) demonstrates a high capacity for screening different compounds with different chemical moieties by using barcode‐like identification patterns. The PCN‐based membranes exhibited high stability, enabling their use for at least 3 months after fabrication.

## 4 Experimental Section

4.1

4.1.1

##### Chemicals

All chemical reagents were of analytical grade, obtained from commercial suppliers, and used without further purification unless otherwise noted. The used reagents were zirconium(IV) butoxide solution (Zr(OBu)_4_), 80 wt% in 1‐butanol; Sigma Aldrich;) tetrakis(4‐carboxyphenyl)porphyrin (TCPP; BLD Pharmatech; 97%); benzoic acid (Sigma Aldrich, 99.5%); N,N‐dimethylformamide anhydrous (DMF; Panreac, 99.8%), methanol (MeOH; Panreac, 99.9%), and 1‐propanol (Panreac, 99.8%). The synthesis of the Zr_6_ nodes was performed following a published method.^[^ ^74^ ^]^ For metalation reactions, the metal salts used as precursors were the following: AgNO_3_, ZnCl_2_, FeCl_2_, CuCl_2_, and CoCl_2_, (>98% in all cases, Sigma‐Aldrich). For membrane preparation, the polymer matrix, poly(dimethylsiloxane), commercialized as Sylgard 184 was purchased from Dow Corning.

##### Characterization

TEM images were acquired using a JEOL 2100Plus operated at 200 kV. Elemental composition was analyzed by EDX using the same TEM instrument with an integrated Oxford INCA EDX system. Copper grids coated with Formvar/carbon film were used for the deposition of the particles, except in the case of Cu‐metalated PCN‐222, for which a nickel grid was used. Elemental mapping was performed by EDX using a TEM FEI TALOS X‐200 instrument. SEM images were acquired with a ZEISS GeminiSEM 300. The particles were deposited on a silicon wafer substrate. XRD of the powdered samples was performed using a Bruker D8‐Advance Diffractometer operated at 50 kV and 1 mA for Cu k*α* (*λ* = 1.5418 Å) in the range 2–5° (2*θ*) with a step of 0.01° 1 s^−1^. In addition, GAXRD was used to determine the crystalline phase of the MOFs in the membranes. The incidence angle was fixed at 1°, and the diffractograms were recorded in the range of 2–15° (2*θ*) with a step of 0.02° 0.5 s^−1^. All measurements were collected in 2D mode using a Bruker EIGER2 R 250 K Detector. A Malvern Zetasizer Nano ZSP equipped with a 10 mW He–Ne laser operating at a wavelength of 633 nm and fixed scattering angle of 173° was used for the DLS measurements. Diluted dispersions of the particles were loaded into a quartz cuvette and equilibrated at room temperature for 2 min. Size distribution results were generated by 3 consecutive measurements (*n* = 3), each consisting of 12 data runs. N_2_ isotherms (77 K) were obtained using a Micromeritics Tristar II 3020 system. Before the adsorption measurements, samples were outgassed under a vacuum at 120 °C for 24 h. The apparent surface areas were calculated from the BET method in the pressure interval *P*/*P*_0_ = 0.01–0.2 (being *P*_0_ the saturation pressure). Micropore volume was calculated using the t‐plot method, while total pore volume was estimated at *P*/*P*_0_ ≈ 0.95. Mesopore volume was estimated from the difference between total and micropore volume. Pore size distribution analysis was carried out using the non‐local density functional theory method. The UV–vis absorbance spectra of diluted suspensions of the MOFs particles and MOF@PDMS films were obtained using an Agilent Cary‐100 spectrophotometer in the 350–700 nm range.

##### MW‐Assisted Synthetic Procedure for Nano‐ and Microsized PCN‐222 Particles

The MW‐assisted synthesis of the PCN‐222 particles was carried out on an Initiator Classic Microwave reactor from Biotage. The procedure for achieving nanosized PCN‐222 particles (≈100 nm in length) was optimized as follows (Scheme S1, Supporting Information). In brief, Zr_6_ clusters, [Zr_6_(μ_3_‐O)_4_(μ_3_‐OH)_4_], (76 mg, 28.4 μmol, dissolved in 4 mL DMF), TCPP (22.5 mg, 28.5 μmol, dissolved in 4 mL DMF), and TFA (100 μL) were placed in a 10 mL MW vial. By applying an initial MW power of 250 W under continuous stirring (600 rpm), the mixture was quickly heated to 100 °C and then maintained at this temperature for 10 min. Afterward, the reaction was quickly cooled down with the aid of cooling air flow to quench the further crystals growth. The resulting PCN‐222 particles were separated by centrifugation (10 000 RCF, 15 min), washed twice with fresh DMF, and then twice with MeOH. Finally, the purified PCN‐222 particles were redispersed in MeOH at a concentration of 5 mg mL^−1^ and these suspensions were stored in the fridge until use. To obtain large PCN‐222 particles under MW irradiation, the temperature and time were increased to 120 °C and 30 min, respectively, and higher amounts of the acid modulator were added (200 μL for 1 μm‐size, and 400 μL for 1.6 μm‐size particles). The purification of the resulting microsized PCN‐222 particles was identical as described above for nanosized PCN‐222.

##### MW‐Assisted Method for the Metalation of PCN‐222

Metalation of the PCN‐222 particles with Ag, Zn, Fe, Cu, and Co, denoted as PCN(Ag), PCN(Zn), PCN(Fe), PCN(Cu), and PCN(Co), was carried out by using an optimized MW‐method (Scheme S1, Supporting Information). In the cases of PCN‐222(Zn), PCN(Fe), and PCN(Cu), 5 mg of the purified PCN‐222 particles were dispersed in 2 mL of MeOH and mixed with the respective metal salts in methanol (0.1 mmol of the metal dissolved in 1 mL of MeOH) in a 10 mL MW vial. The mixture was heated to 80 °C for 15 min by applying an initial MW power of 150 W and under continuous stirring (600 rpm); lower reaction times did not lead to complete metalation. After this time, the reaction was let to cool down to room temperature, and the resulting particles were separated by centrifugation (10 000 RCF, 15 min). The particles were washed twice with methanol and finally redispersed in MeOH at a concentration of 5 mg mL^−1^. For the preparation of PCN(Ag) and PCN(Co), the same procedure was performed but using DMF as the reaction solvent instead of MeOH.

##### Membrane Fabrication

100 μL of a 5 mg mL^−1^ PCN suspension in methanol was mixed with Sylgard™ 184 base and a curing agent in a 10:1 wt ratio (1 and 0.1 g, respectively). The mixture was vigorously stirred for ≈1 h. After the complete dispersion of the MOF particles into the polymer matrix, the methanol was removed by rotatory evaporation. Then, the mixture was degassed applying low vacuum and, subsequently, spin‐coated on a Petri dish at 1000 rpm for 60 s. Finally, the Petri dish was placed in an oven at 60 °C overnight to cure the polymer phase. The films were cut and peeled off on demand.

##### Sensing Assays

The different PCN‐modified PDMS films were exposed to saturated vapors oftwelve analytes, i.e., 2,4‐dinitrotoluene (DNT), acetone, chloroform, N‐butylamine, tetrahydrofuran, hydrogen sulfide, hydrogen chloride, dichloromethane, ammonia, hexanal, toluene, and ethanol. The saturated vapors of each VOC were obtained by bubbling dry N_2_ through the pure liquid analyte at room temperature, resulting in a mixture of N_2_ saturated with each VOC. The gas flow rates were controlled using two Bronkhorst F‐201FV mass flow controllers and the gas flow rate was fixed at 0.2 L min^−1^. The gas mixture was then introduced into a sealed vial where the samples had previously been placed for exposure. The UV–vis absorption spectra of the PCN@PDMS and PCN(M)@PDMS (M = Fe, Co, Cu, Zn, and Ag) films were recorded before and after 30 min exposure. For the sensing measurements of DNT, the exposure was carried out as follows: PDMS films were placed in a glass vial that had been saturated with vapors of solid explosive (≈10 mg) and hermetically sealed. The exposure was continued for 48 h to ensure complete saturation.

## Conflict of Interest

The authors declare no conflict of interest.

## Author Contributions

**Francisco G. Moscoso**: Conceptualization (equal); Formal analysis (equal); Investigation (equal); Methodology (equal); Writing—original draft (lead); Writing—review & editing (equal). **Juan J. Romero‐Guerrero**: Formal analysis (equal); Investigation (equal). **David Rodriguez‐Lucena**: Investigation (equal); Methodology (equal). **José María Pedrosa**: Conceptualization (lead); Funding acquisition (lead); Investigation (lead); Project administration (lead); Supervision (lead); Writing—review & editing (lead). **Carolina Carrillo‐Carrión**: Data curation (lead); Funding acquisition (equal); Investigation (lead); Methodology (lead); Resources (equal); Supervision (equal); Writing—original draft (equal); Writing—review & editing (equal).

## Supporting information

Supplementary Material

## Data Availability

The data that support the findings of this study are available in the supplementary material of this article.
