# Supplementary material for: Nanosized Porphyrinic Metal–Organic Frameworks for the Construction of Transparent Membranes as a Multiresponsive Optical Gas Sensor
Source: Small Sci. 2024 Aug 19;4(10):2400210. doi: 10.1002/smsc.202400210 (PMC11935058; doi:10.1002/smsc.202400210)
Supplement: Supplementary file 1 — Supplementary Material [file SMSC-4-2400210-s001.zip › smsc.202400210-sup-0001-suppdata-S1.pdf]

Supporting Information

Nanosized Porphyrinic Metal–Organic Frameworks for the Construction of Transparent Membranes as a Multi-Responsive Optical Gas Sensor

Francisco G. Moscoso, Juan J. Romero-Guerrero, David Rodriguez-Lucena, José M. Pedrosa , and Carolina Carrillo-Carrión**

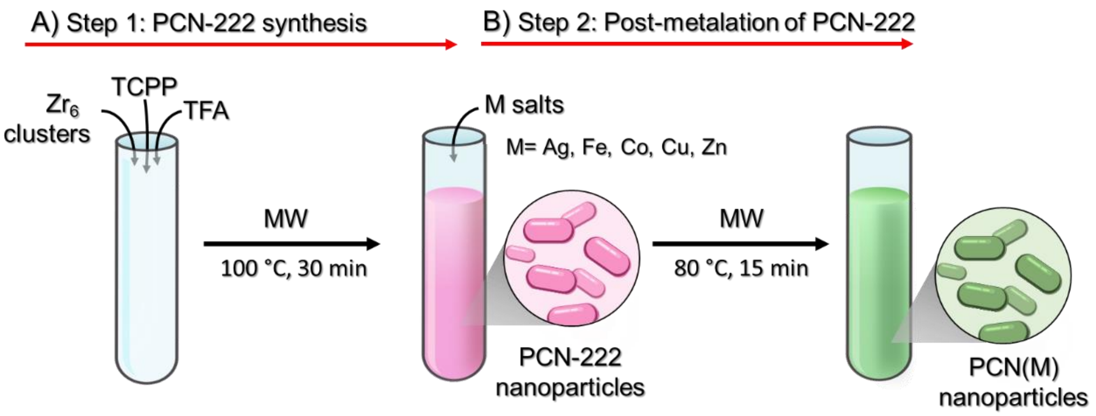

Scheme S1. (A) MW-assisted synthesis of PCN-222 nanoparticles by reaction of TCPP linkers with Zr_6 nodes and using TFA as a modulator. (B) Post-synthetic metalation of the PCN-222 nanoparticles under MW irradiation.

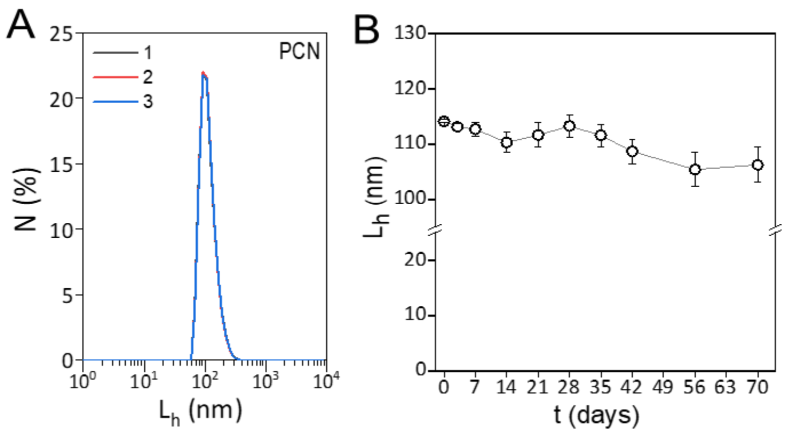

Figure S1. (A) DLS size distributions by number of the PCN-222 nanoparticles dispersed in methanol (n=3, mean hydrodynamic length of L_h=114 nm). (B) Colloidal stability over time of the PCN-222 nanoparticles dispersed in methanol as determined by DLS.

Table S1. Textural properties of PCN-222 as obtained from N_2 sorption analysis.

Sample	S_{BET} ($m^2 \cdot g^{-1}$)	V_{micro} ($cm^3 \cdot g^{-1}$)	V_{meso} ($cm^3 \cdot g^{-1}$)
PCN-222	2108	0.68	0.87

Surface area (S_{BET}) calculated by BET equation; total volume (V_{total}) determined at P/P_0 =0.95; micropore volume (V_{micro}) calculated by t-plot method; mesopore volume (V_{meso}) calculated from V_{total}-V_{micro}.

Table S2. Hydrodynamic sizes L_h (mean ± SD) at different time points as derived from DLS number distributions of the PCN-222 nanoparticles dispersed in methanol. Data correspond to the raw data shown in Fig. S1.

Time (day)	L_h ± SD (nm)	PDI
0	114 ± 0.3	0.081
3	113 ± 0.7	0.097
7	113 ± 1.2	0.101
14	110 ± 1.8	0.108
21	112 ± 2.2	0.103
28	113 ± 2.1	0.105
35	111 ± 2.0	0.108
42	108 ± 2.2	0.134
56	105 ± 3.1	0.141
70	106 ± 3.2	0.150

Table S3. Miller indices (hkl), reflection angles (2θ) and d-space of the first ten reflection peaks of the diffractogram in Figure 1.

(hkl)	2θ (º)	d (Å)
100	2.36	37.34
110	4.13	21.33
200	4.79	18.42
001	5.12	17.22
101	5.62	15.59
111	6.65	13.28
201	7.06	12.5
300	7.2	12.08
211	8.2	10.75
400	9.77	9.04

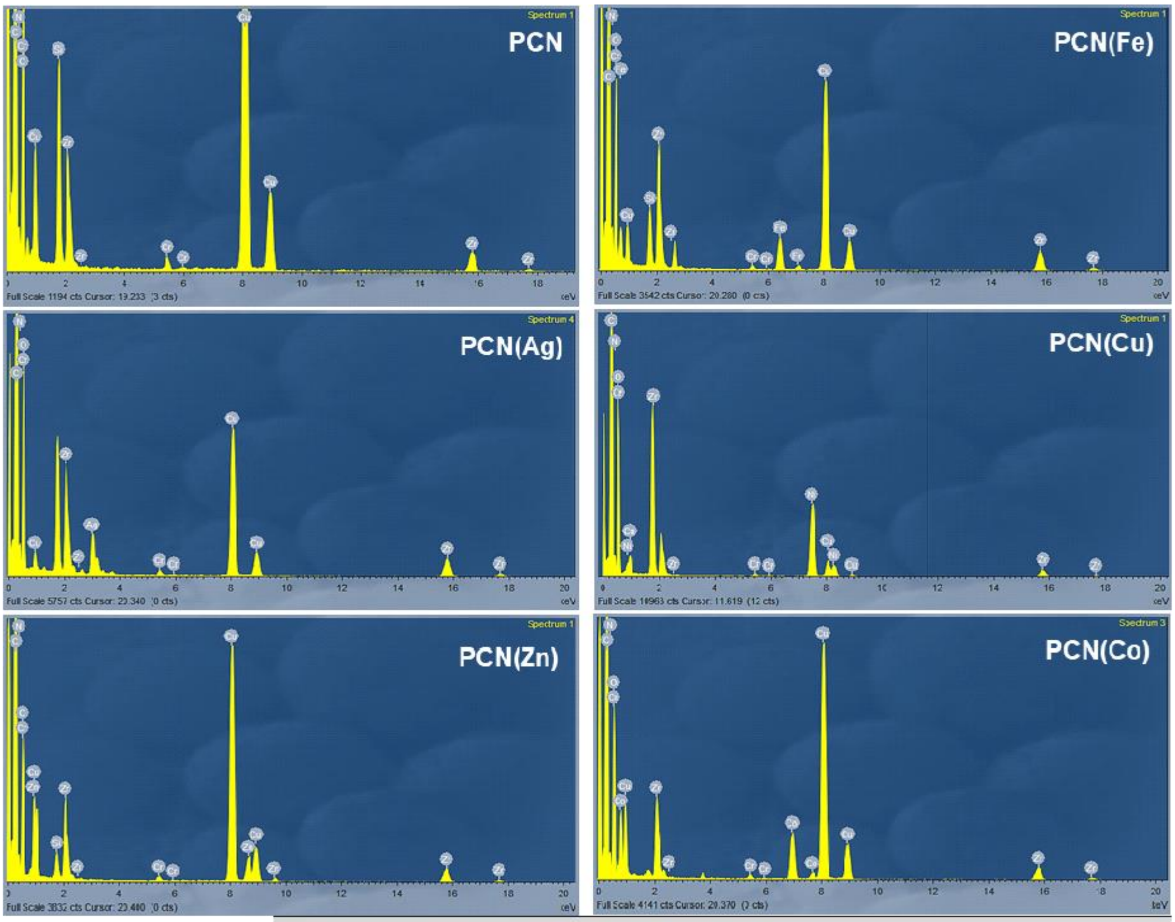

	PCN(Ag)	PCN(Zn)	PCN(Fe)	PCN(Cu)	PCN(Co)
**M/Zr ratio (mol%)**	0.44	0.47	0.42	0.42	0.48

Figure S2. TEM-EDX analyses of the metalated PCN(M) nanoparticles, indicating the obtained atomic molar Zr/M ratio in each case.

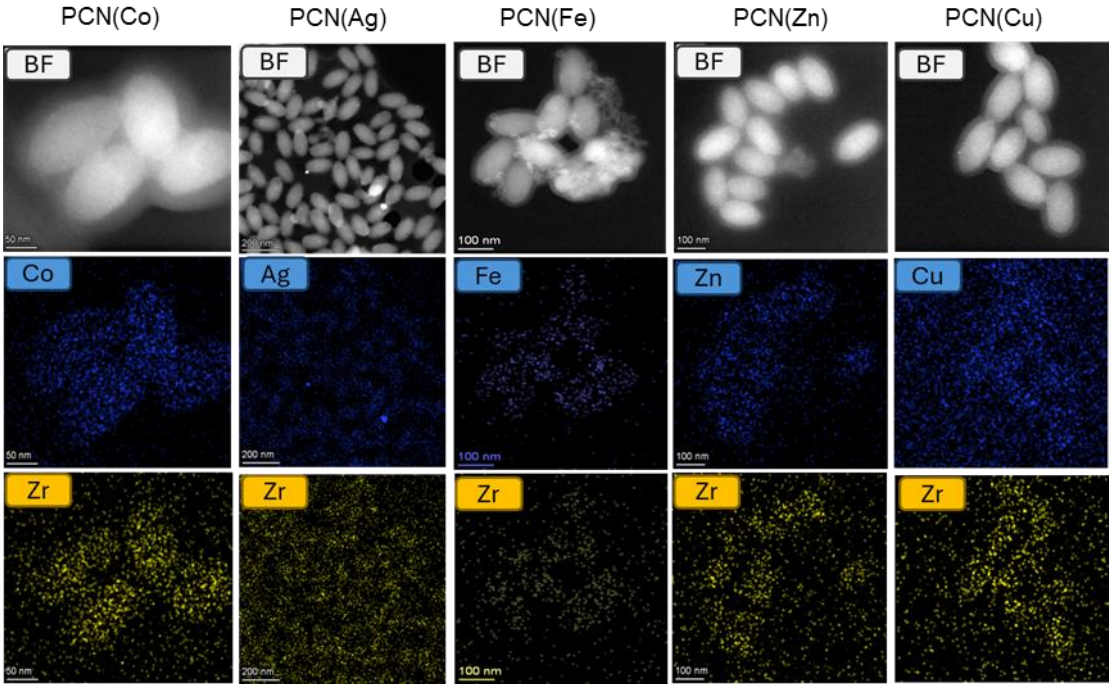

Figure S3. EDS elemental mapping of the different metalated PCN(M) nanoparticles.

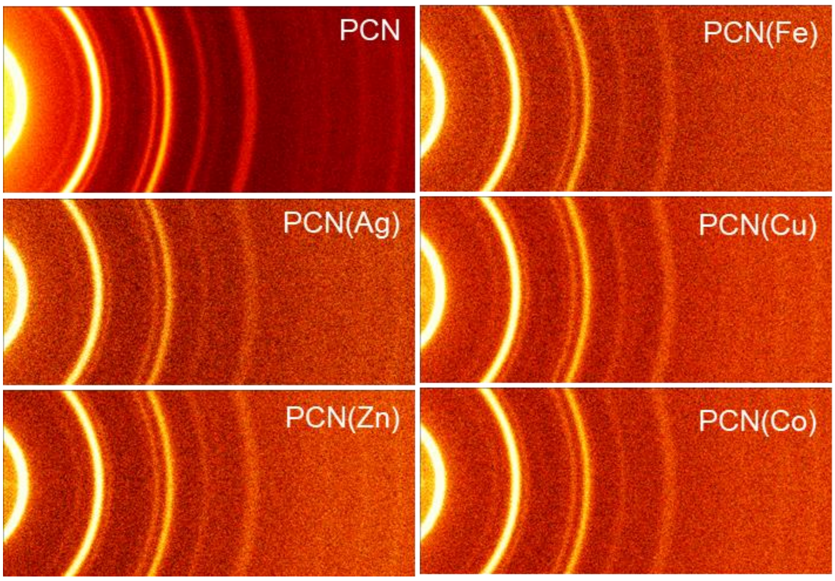

Figure S4. 2D XRD of the different metalated PCN(M) nanoparticles compared with the pristine non-metalated PCN-222.

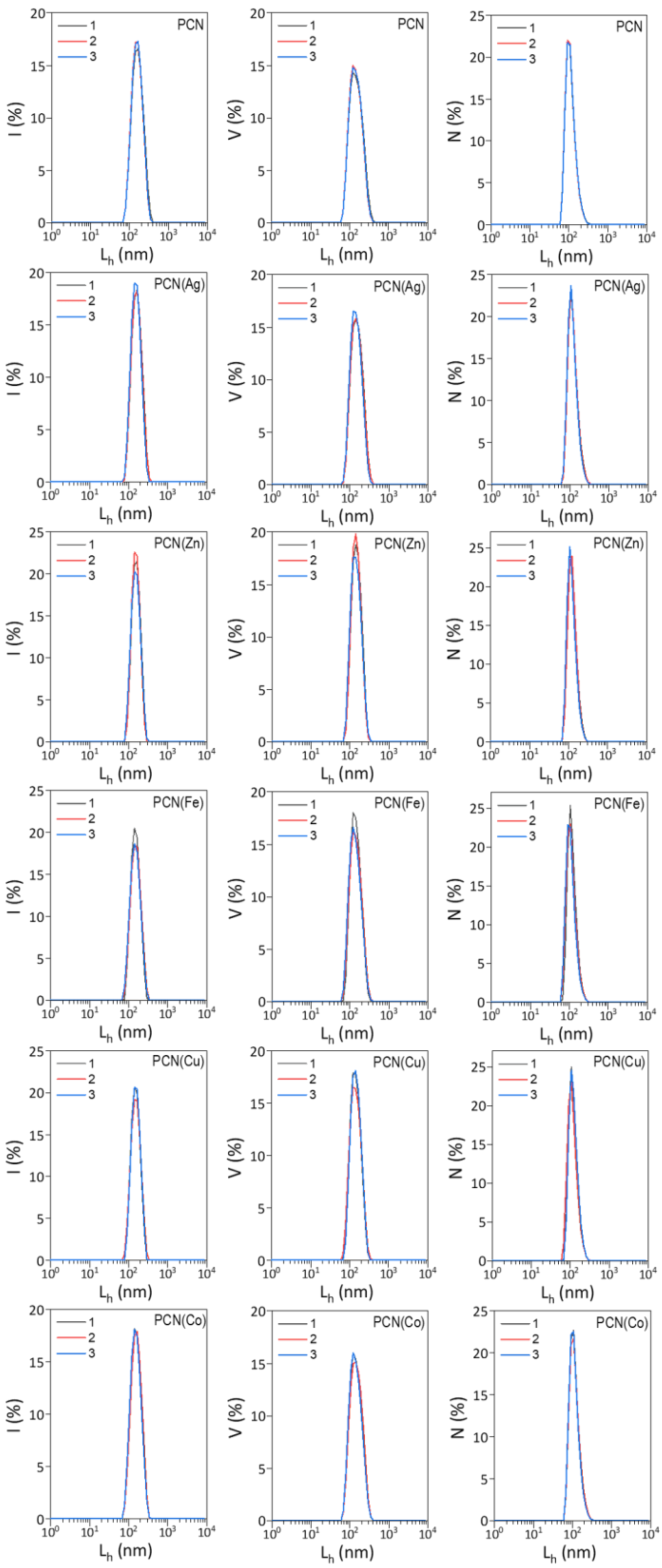

Figure S5. DLS intensity (I), volume (V), and number (N) distributions of the hydrodynamic sizes (L_h) of the non-metalated and the different metalated PCN(M) nanoparticles (M=Ag, Zn, Fe, Cu, and Co) as dispersed in methanol.

Table S4. Hydrodynamic sizes of the non-metalated and the different metalated PCN(M) nanoparticles (M=Ag, Zn, Fe, Cu, and Co) as dispersed in methanol. Values are expressed as mean hydrodynamic length ± standard deviation (L_h ± SD) and the polydispersity index (PDI) for each sample is also shown. Data correspond to number distribution curves shown in Fig. S4.

	**PCN-222**	**PCN(Ag)**	**PCN(Zn)**	**PCN(Fe)**	**PCN(Cu)**	**PCN(Co)**
**L_h ± SD (nm)**	114 ± 0.3	122 ± 2.2	125 ± 2.9	116 ± 4.1	121 ± 3.1	115 ± 1.4
**PDI**	0.081	0.094	0.072	0.086	0.075	0.085

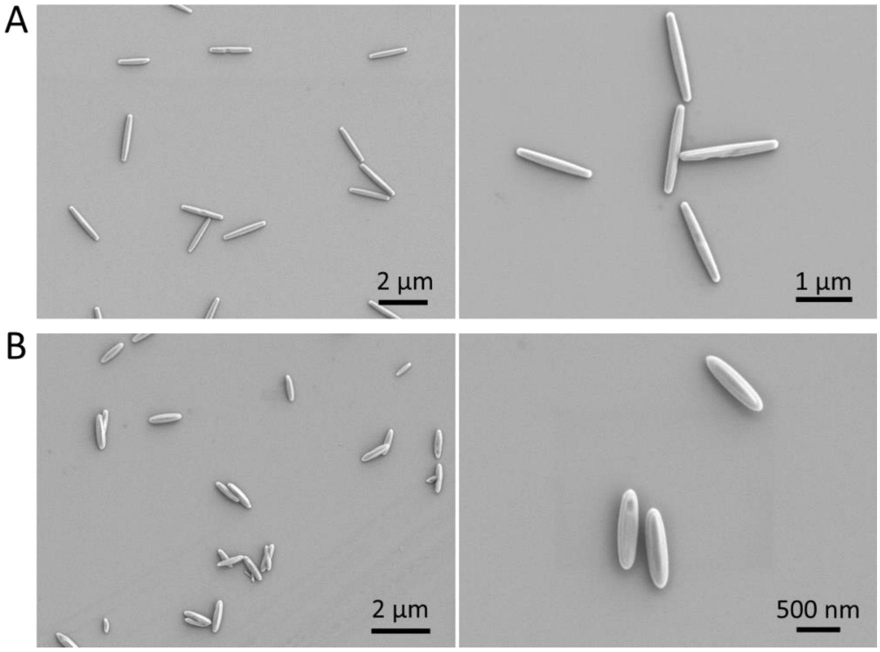

Figure S6. SEM images of microsized PCN-222 particles having an average length of (A) 1.6 µm, and (B) 1 µm.

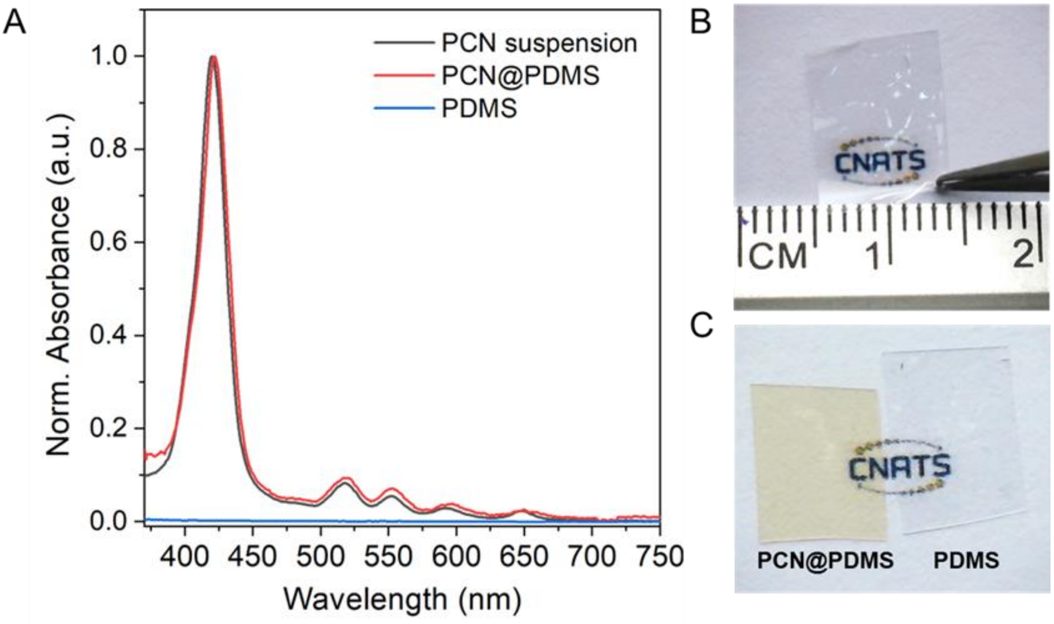

Figure S7. (A) Normalized absorbance spectra of a PCN-222 suspension (black), PCN@PDMS membrane (red), and PDMS membrane (blue) (B) Photographs of the PDMS membrane, (C) visual comparison of PCN@PDMS and PDMS membranes.

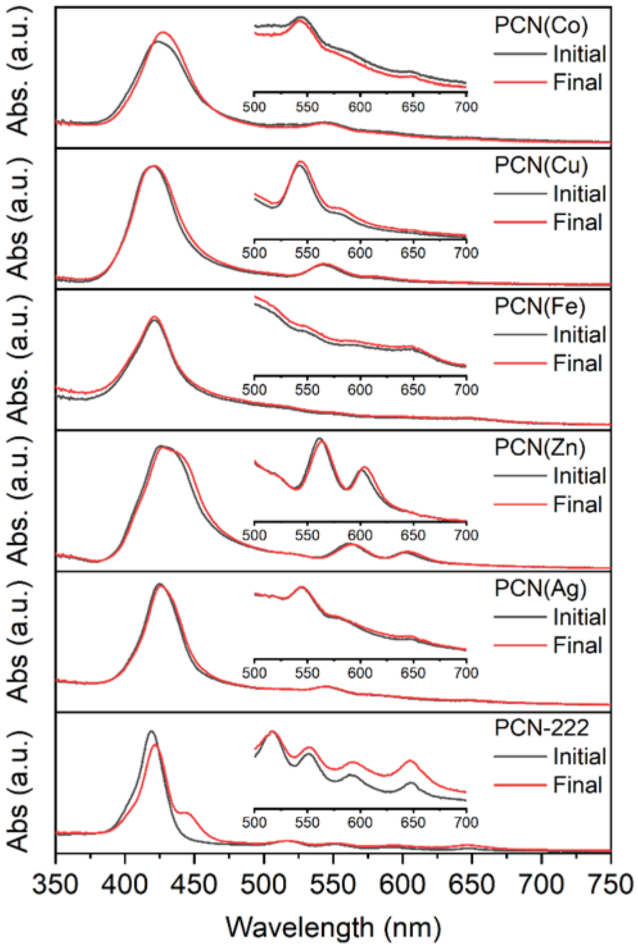

Figure S8. UV-Vis spectra of ethanol suspensions of PCN-222 and PCN(M) before (black line) and after (red line) TFA addition (TFA:porphyrin molar ratio 10:1).

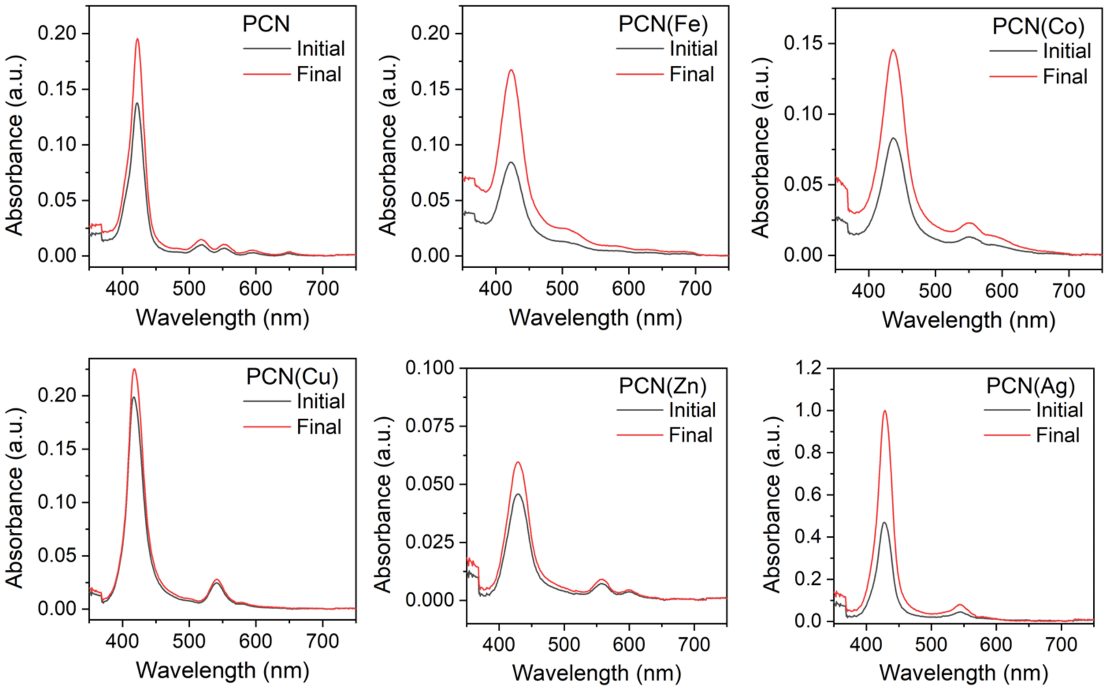

Figure S9. Absorbance spectra of PCN@PDMS and PCN(M)@PDMS before and after the exposure to saturated vapors of acetone.

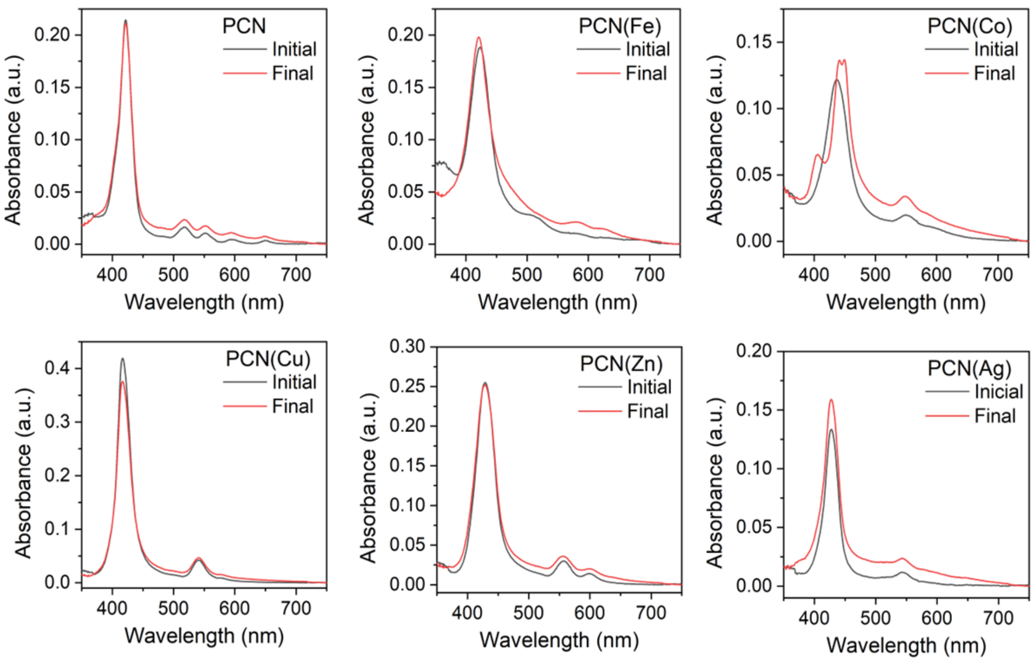

Figure S10. Absorbance spectra of PCN@PDMS and PCN(M)@PDMS before and after the exposure to saturated vapors of ammonia.

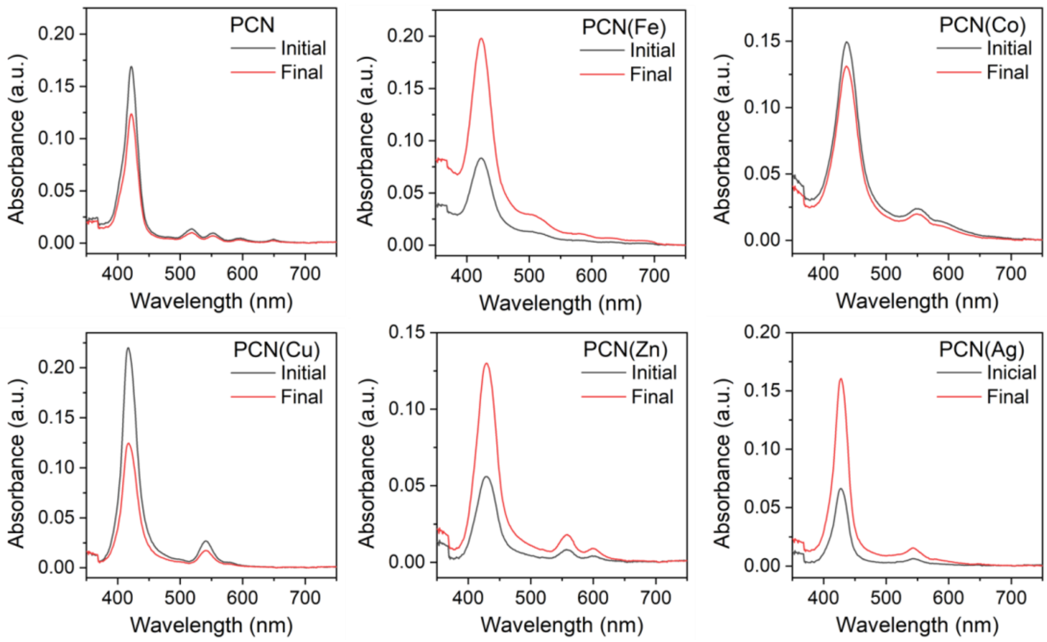

Figure S11. Absorbance spectra of PCN@PDMS and PCN(M)@PDMS before and after the exposure to saturated vapors of chloroform.

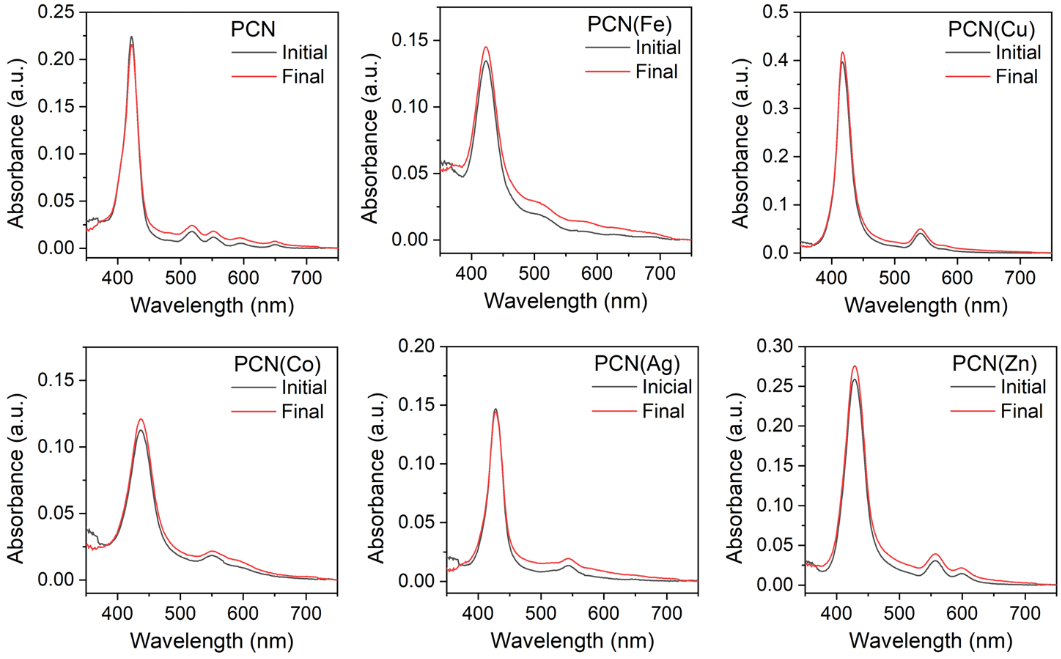

Figure S12. Absorbance spectra of PCN@PDMS and PCN(M)@PDMS before and after the exposure to saturated vapors of dichloromethane.

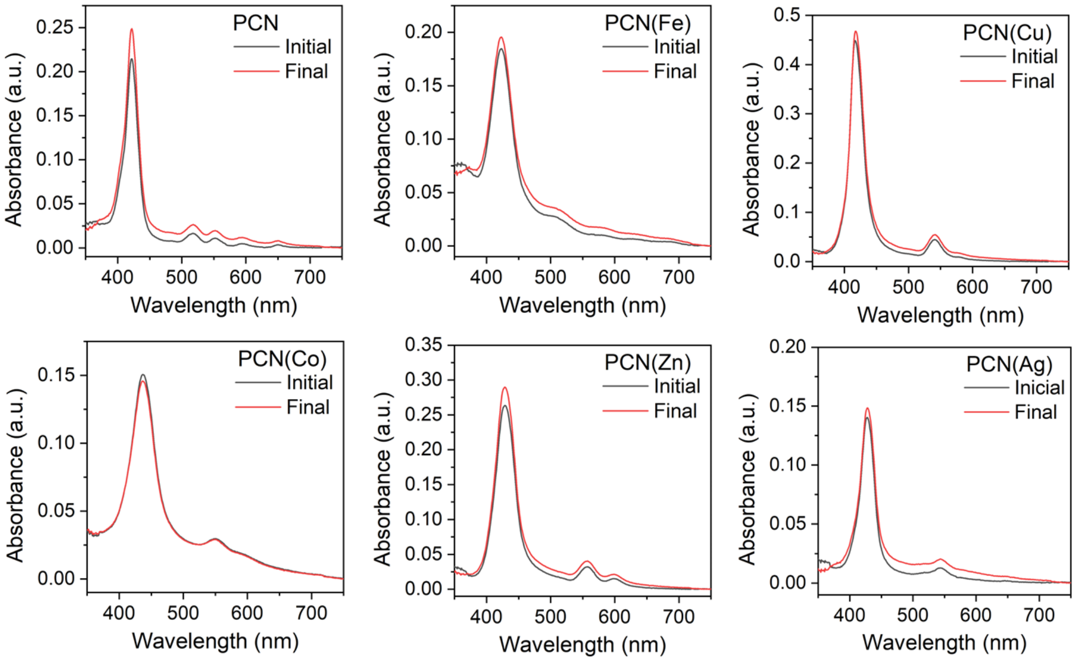

Figure S13. Absorbance spectra of PCN@PDMS and PCN(M)@PDMS before and after the exposure to saturated vapors of ethanol.

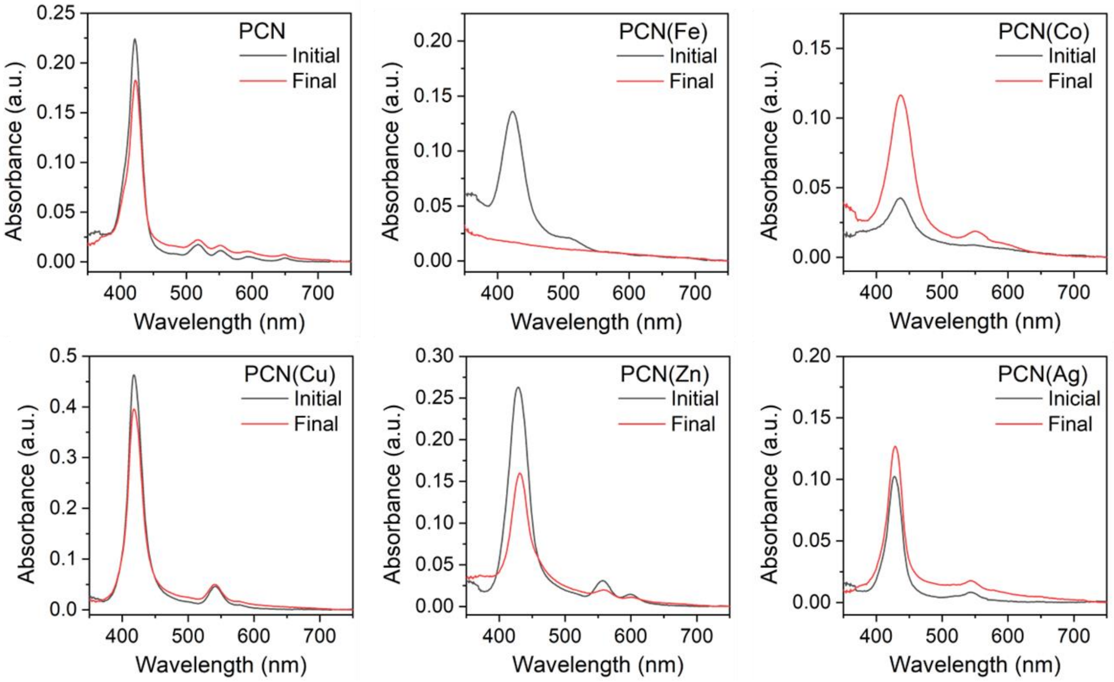

Figure S14. Absorbance spectra of PCN@PDMS and PCN(M)@PDMS before and after the exposure to saturated vapors of hexanal.

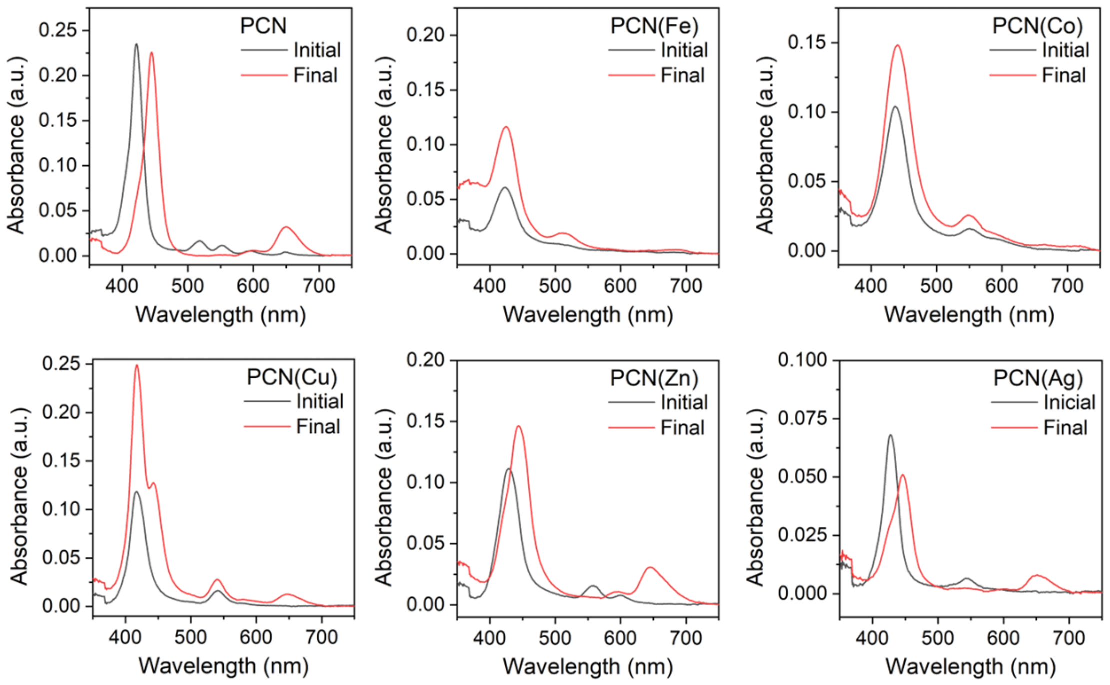

Figure S15. Absorbance spectra of PCN@PDMS and PCN(M)@PDMS before and after the exposure to saturated vapors of hydrogen chloride.

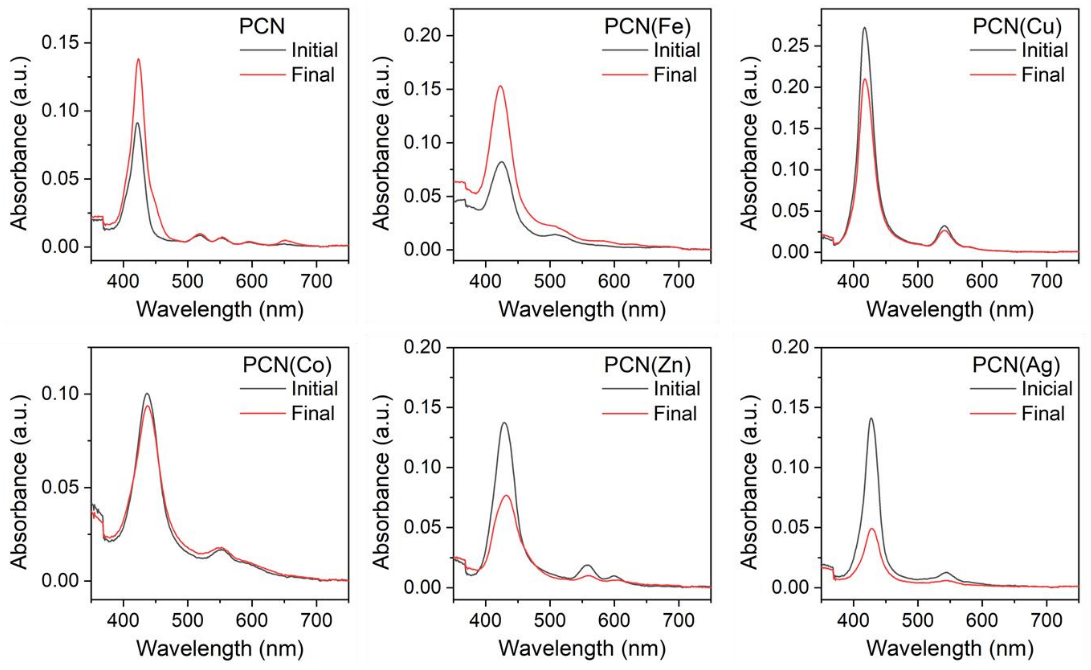

Figure S16. Absorbance spectra of PCN@PDMS and PCN(M)@PDMS before and after the exposure to saturated vapors of hydrogen sulfide.

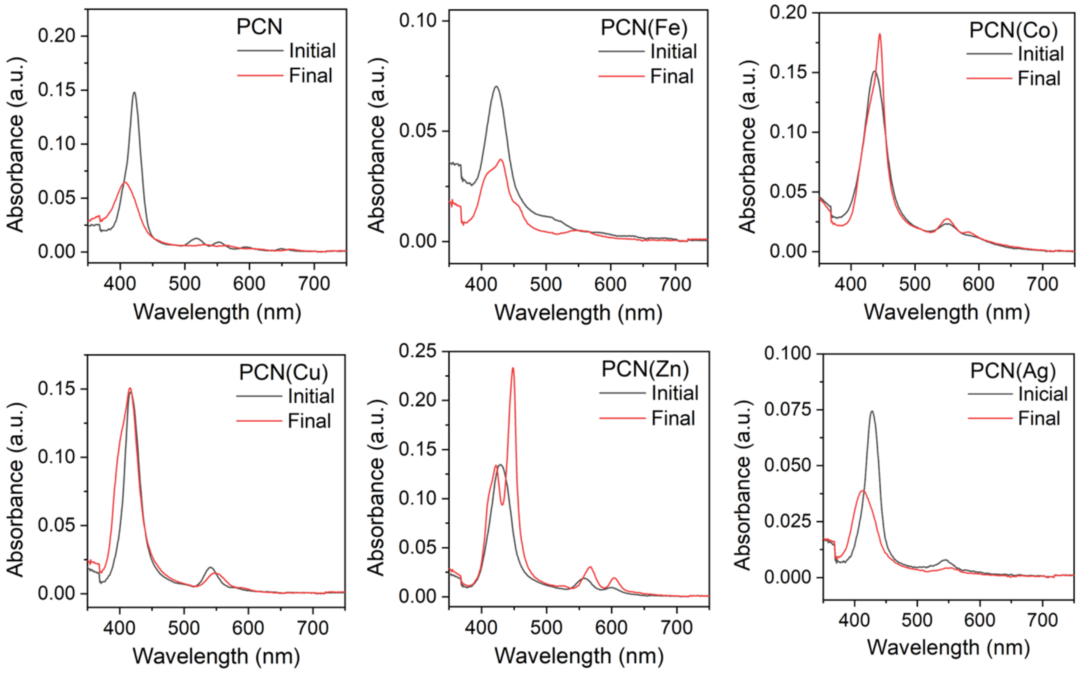

Figure S17. Absorbance spectra of PCN@PDMS and PCN(M)@PDMS before and after the exposure to saturated vapors of N-butylamine.

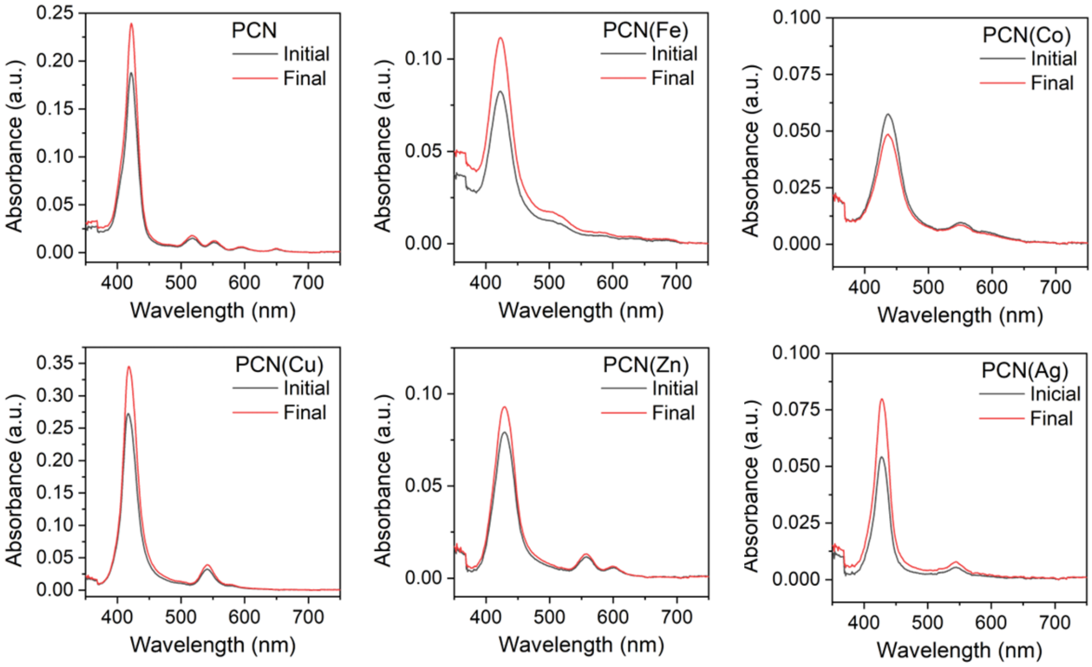

Figure S18. Absorbance spectra of PCN@PDMS and PCN(M)@PDMS before and after the exposure to saturated vapors of tetrahydrofuran.

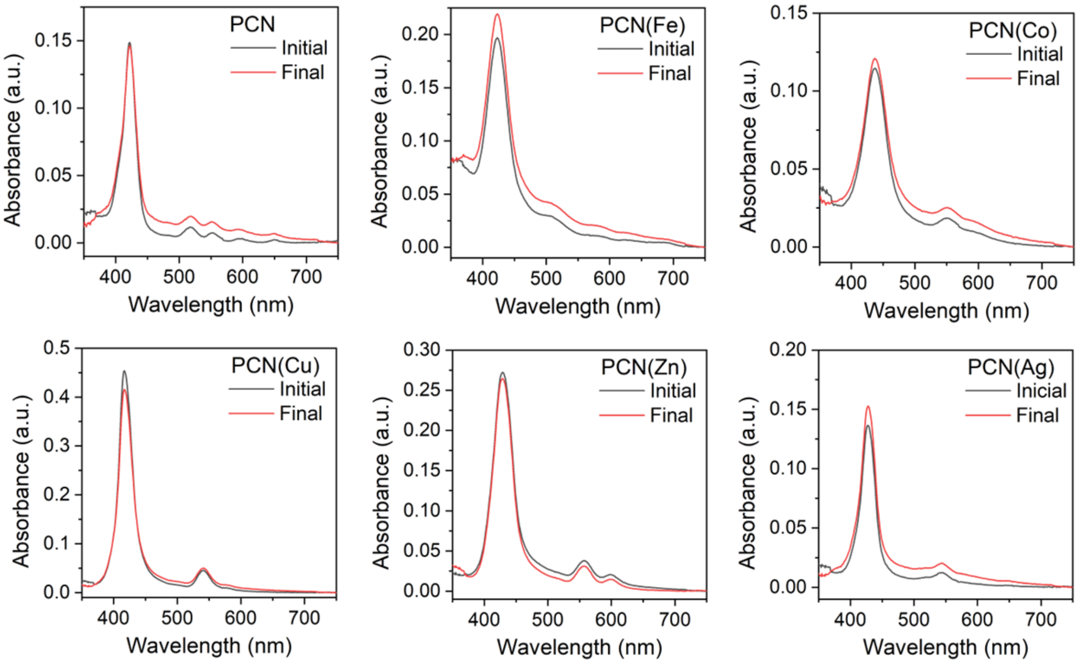

Figure S19. Absorbance spectra of PCN@PDMS and PCN(M)@PDMS before and after the exposure to saturated vapors of toluene.

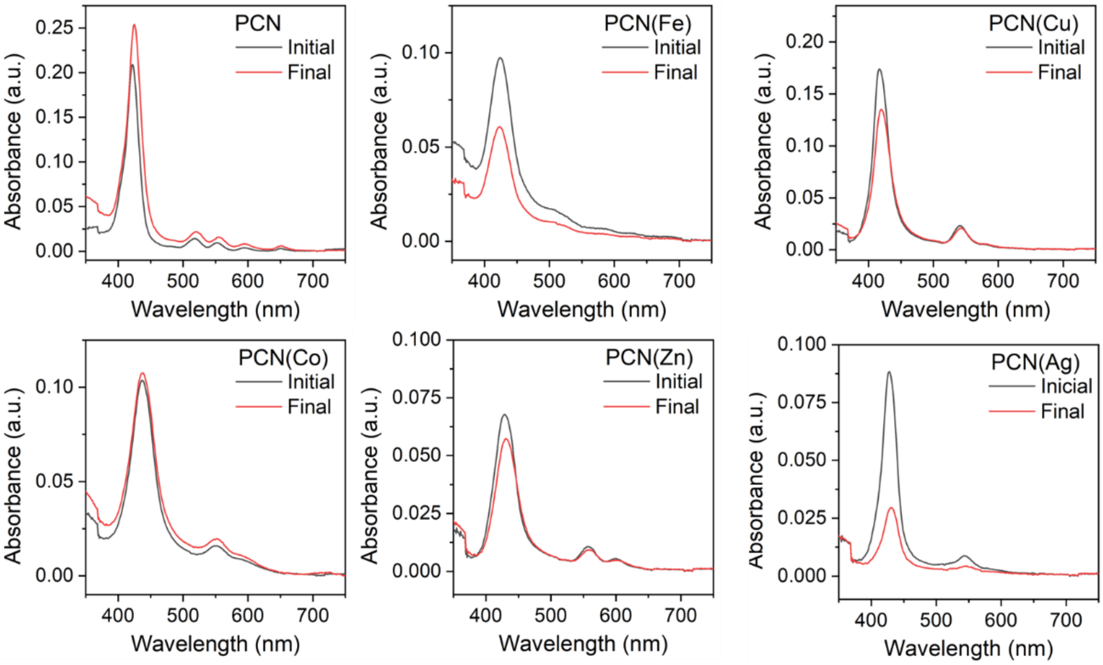

Figure S20. Absorbance spectra of PCN@PDMS and PCN(M)@PDMS before and after the exposure to saturated vapors of 2,4-dinitrotoluene.

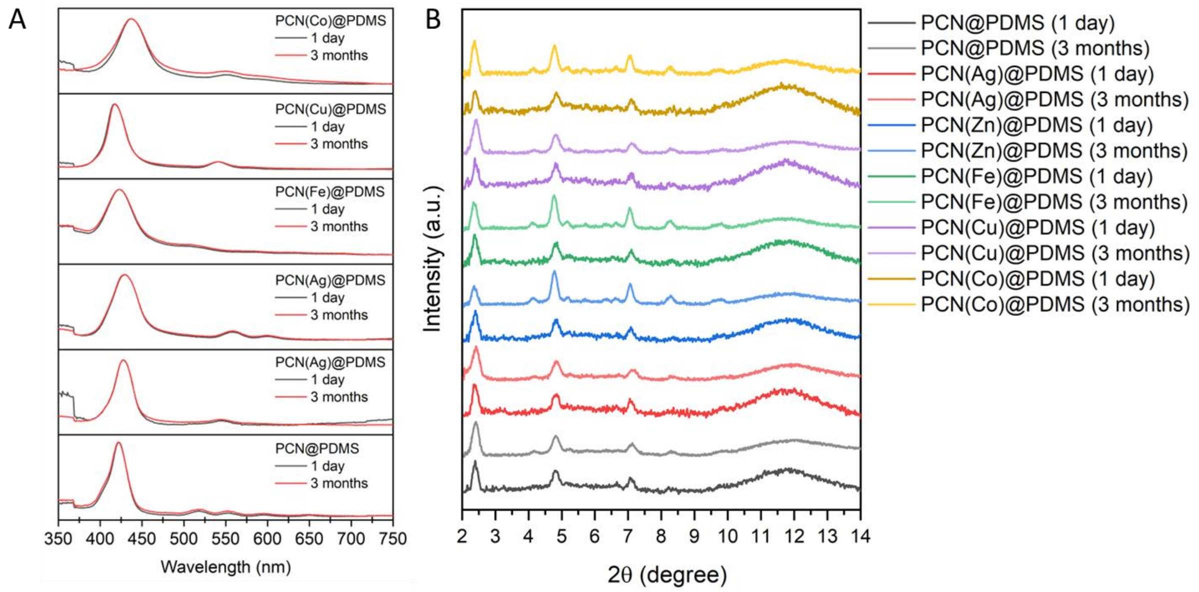

Figure S21. (A) UV-Vis spectra of PCN@PDMS and PCN(M)@PDMS films after 1 day (black line) and after 3 months (red line) from membrane fabrication. (B) GAXRD of PCN@PDMS and PCN(M)@PDMS films after 1 day and 3 months from the fabrication.

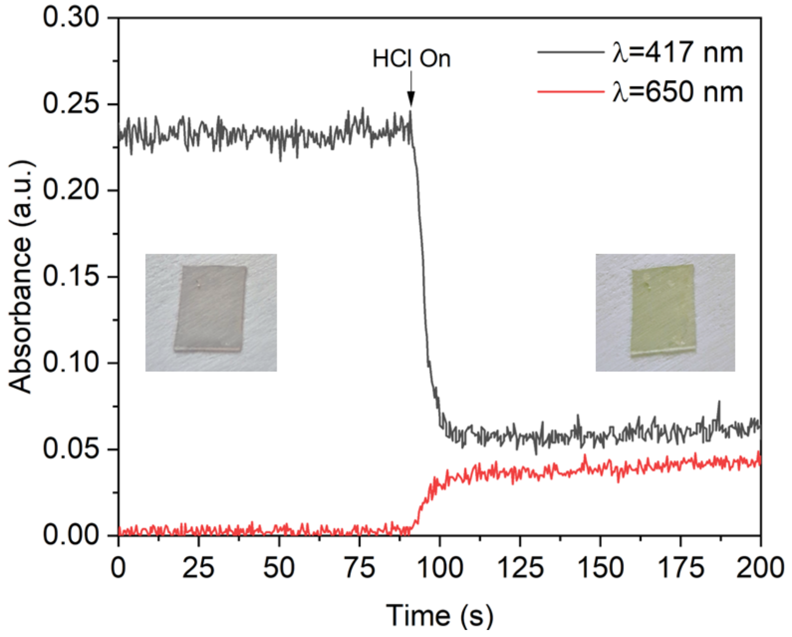

Figure S22. Temporal evolution of the absorbance at 417 nm (black line) and 650 nm (red line) of a PCN@PDMS membrane under exposure to HCl vapors. Inset: Photographs of the film before and after exposure.
